# Platinum Nanozymes Counteract Photoreceptor Degeneration and Retina Inflammation in a Light-Damage Model of Age-Related Macular Degeneration

**DOI:** 10.1021/acsnano.3c07517

**Published:** 2023-11-07

**Authors:** Sara Cupini, Stefano Di Marco, Luca Boselli, Alessio Cavalli, Giulia Tarricone, Valentina Mastronardi, Valentina Castagnola, Elisabetta Colombo, Pier Paolo Pompa, Fabio Benfenati

**Affiliations:** † Center for Synaptic Neuroscience and Technology, Istituto Italiano di Tecnologia, Largo Rosanna Benzi 10, 16132 Genova, Italy; ‡ Department of Experimental Medicine, University of Genova, Viale Benedetto XV 3, 16132 Genova, Italy; ∥ Nanobiointeractions & Nanodiagnostics, Istituto Italiano di Tecnologia, Via Morego 30, 16163 Genova, Italy; § IRCCS Ospedale Policlinico San Martino, Largo Rossana Benzi 10, 16132 Genova, Italy

**Keywords:** oxidative stress, nanoparticles, photoreceptor death, Müller cells, microglia, electroretinogram, high-density multielectrode arrays

## Abstract

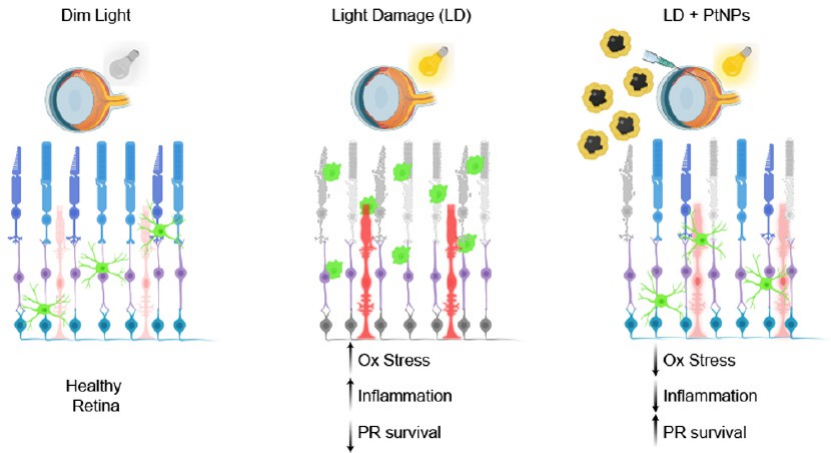

Degeneration of photoreceptors in age-related macular degeneration (AMD) is associated with oxidative stress due to the intense aerobic metabolism of rods and cones that if not properly counterbalanced by endogenous antioxidant mechanisms can precipitate photoreceptor degeneration. In spite of being a priority eye disease for its high incidence in the elderly, no effective treatments for AMD exist. While systemic administration of antioxidants has been unsuccessful in slowing down degeneration, locally administered rare-earth nanoparticles were shown to be effective in preventing retinal photo-oxidative damage. However, because of inherent problems of dispersion in biological media, limited antioxidant power, and short lifetimes, these NPs are still confined to the preclinical stage. Here we propose platinum nanoparticles (PtNPs), potent antioxidant nanozymes, as a therapeutic tool for AMD. PtNPs exhibit high catalytic activity at minimal concentrations and protect primary neurons against oxidative insults and the ensuing apoptosis. We tested the efficacy of intravitreally injected PtNPs in preventing or mitigating light damage produced in dark-reared albino Sprague–Dawley rats by *in vivo* electroretinography (ERG) and *ex vivo* retina morphology and electrophysiology. We found that both preventive and postlesional treatments with PtNPs increased the amplitude of ERG responses to light stimuli. *Ex vivo* recordings demonstrated the selective preservation of ON retinal ganglion cell responses to light stimulation in lesioned retinas treated with PtNPs. PtNPs administered after light damage significantly preserved the number of photoreceptors and inhibited the inflammatory response to degeneration, while the preventive treatment had a milder effect. The data indicate that PtNPs can effectively break the vicious cycle linking oxidative stress, degeneration, and inflammation by exerting antioxidant and anti-inflammatory actions. The increased photoreceptor survival and visual performances in degenerated retinas, together with their high biocompatibility, make PtNPs a potential strategy to cure AMD.

## Introduction

Rod and cone photoreceptors (PRs) in the retina are characterized by the highest oxidative metabolic rate in the body. They are exposed to a very large oxygen gradient that, highest in the choroid, sharply falls in the outer retina devoid of a direct blood supply. In addition, PRs are continuously exposed to light that can induce phototoxicity above a certain photon power density. These lifelong conditions make PRs extremely vulnerable to oxidative damage and to the deleterious effects of reactive oxygen species (ROS). When these species, primarily generated by the electron transport chain in the mitochondria, rise above the low physiological levels (<100 nM),^1^ progressive cell damage occurs, with alterations in DNA, cell membranes, and intracellular signaling pathways, triggering cell death programs and inflammatory processes.^2−4^ Under physiological conditions, the effects of the oxidative microenvironment are neutralized by endogenous antioxidant and cell repair systems in the retinal pigment epithelium (RPE) and PRs. However, when mutations of genes critical for these antioxidant activities or prolonged exposure to the oxidative milieu during aging occur, PR degeneration takes place, leading to diseases such as retinitis pigmentosa (RP) and atrophic age-related macular degeneration (AMD), respectively. While RP, a collective name for a set of rare genetic disorders that cause the death of rods and secondarily of cones, afflicts 1 in 4000 people worldwide, AMD, defined by the World Health Organization as “*priority eye disease*”, affects about 20% of the population between 70 and 90 years.^5−9^ AMD primarily targets perifoveal rods and foveal cones in the *fovea centralis*, the area responsible for sharp central vision.^10,11^ This strong impairment is devastating in the elderly, leading to cognitive decline and depressive states.^12^ Except for the rare genetic forms, there is a wide consensus that chronic oxidative stress and the ensuing inflammation are key mechanisms involved in the pathogenesis and progression of atrophic AMD.^13−16^

In spite of very high AMD prevalence, therapeutic strategies have not yet proved successful.^17^ Planar retinal prosthetic devices^18,19^ or optogenetics^20,21^ obtained poor results in restoring the high-resolution central vision lost in AMD.^22,23^ Replacement therapies with stem cells, allowing for the differentiation of retinal cells, including RPE and PRs, are promising, although mostly in the preclinical stage.^24−26^ Pharmacological approaches to reduce the rate of disease progression include drugs with antioxidant properties, inhibitors of the complement cascade, and neuroprotective agents. However, no treatment can halt or reverse any stage of dry disease.^27,28^

Nanoparticle (NP)-based therapy has recently been attracting tremendous interest as a potential treatment for neurodegenerative diseases. With respect to parenteral therapies, NPs can be locally injected in the vitreous as colloidal suspensions with ease of administration and high diffusibility. NP-based approaches for preventing or slowing down PR degeneration were explored using titanium dioxide,^29^ silica,^30^ gold,^31,32^ or ceria.^33^ In this context, the downregulation of noxious oxidative stress levels is a promising therapeutic approach. Nanozymes, which are catalytic NPs able to mimic the behavior of natural enzymes, including antioxidant enzymes, have been recently shown to be particularly attractive in this area.^34−37^ Nanozymes hold several advantages compared to their natural counterparts, such as ease of synthetic process, cost-effective scale-up production, high stability, and durability in a wide range of environmental conditions.^38^ Among others, specific types of rare-earth NPs, such as ceria NPs, have been considered for some years as potential therapeutics for ocular diseases.^33,39,40^ For example, nanoceria were demonstrated to prevent PR damage in the retina induced by light damage in dark-reared albino rats and to decrease the extent of degeneration and visual impairment if administered after the damage.^41^ In the last years, nanoceria has been widely applied in various models of oxidative-based degeneration, including light damage^42,43^ or genetic models of the Usher syndrome (*tubby* mouse), retinitis pigmentosa (Rhodopsin^P23H^ rat), or wet AMD^44−47^ and found to be effective also in decreasing the degeneration-induced inflammation. Despite their potential, nanoceria clinical applications are still limited by some concerns related to their potential toxicity.^33^ As an alternative to nanoceria, yttrium NPs have also been proposed with similar performances.^48^

Recently, platinum-based nanoparticles (PtNPs) have emerged as powerful nanozymes, presenting intrinsic, multiple-enzymatic activity, mimicking the main natural antioxidant enzymes such as peroxidase (POD), catalase (CAT), and superoxide dismutase (SOD). The active catalytic sites of PtNPs are their surface atoms, which have significant and persistent ROS-scavenging activities *in vitro* with high performance without the need for specially prepared ligands.^38,49−51^ Fully biocompatible, biocorona-coated PtNPs are endocytosed by cells, whose lysosomes free the NPs from the corona and boost their nanozyme performance thanks to the acidic pH.^51^ Moreover, PtNPs have shown superior stability and biocompatibility *in vitro* and *in vivo*(51) and are emerging for their strong anti-inflammatory potential.^4^ Thus, their application as s nanozyme-based antioxidant therapy for the treatment of a variety of oxidative stress-induced diseases, including neurodegeneration,^52,53^ is very attractive, although they have never been tested *in vivo* as therapeutics for ocular diseases.

In this work, we leveraged on a rat model of AMD generated by a hotspot of PR degeneration in the dorsal retina induced by light damage in dim-light-reared albino rats^54,55^ to investigate the efficacy of the intravitreal administration of PtNPs in either preventing or ameliorating the photo-oxidative retinal damage by *in vivo* electroretinography (ERG) and *ex vivo* retinal morphology and epiretinal recordings by high-density multielectrode arrays (HD-MEA). We found that PtNPs preserved retinal ERG activity, number of PRs, and ON retinal ganglion cell (RGC) responses and mitigated the inflammatory response to degeneration in both preventive and curative protocols, with higher efficacy in the postlesional treatment. The data indicate that low concentrations of PtNPs can effectively break the vicious cycle among ROS, degeneration, and inflammation, making them a potential strategy to cure AMD.

## Results and Discussion

### Platinum Nanoparticles Act as Nanozymes Inducing a Potent and Long-Lasting Scavenging of Reactive Oxygen Species

**Figure 1:**
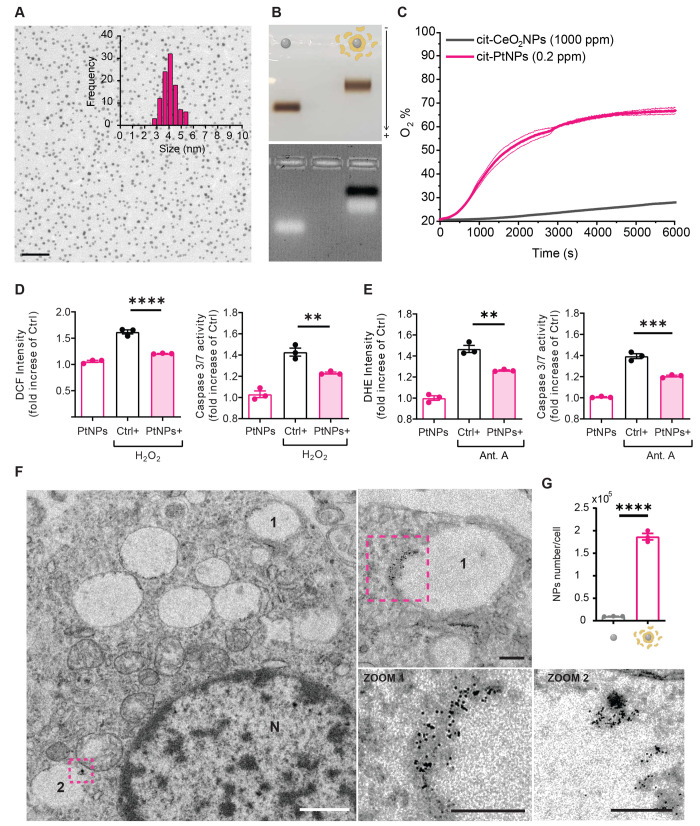
Physical–chemical and biological characterization of platinum nanozymes. (A) Representative TEM micrograph of RSA-coated PtNPs (scale bar, 50 nm) and statistical size analysis (at least 200 NPs were counted). (B) Gel-shift assay in 2.5% agarose gel. The gels are shown in bright-field (top) and UV-transillumination (bottom) modes. In the first lane, the electrophoretic run of citrate-stabilized PtNPs is shown. In the second lane, a delayed electrophoretic run of the RSA-coated PtNPs can be observed. In the transillumination mode, the excess of unbound RSA (black band) is visible. (C) Evaluation of CAT-like activity through overtime monitoring of the oxygen gas developed by the reaction (see Methods). In a vial presenting 20% O_2_ (air), colloidal suspensions of citrate-stabilized PtNPs (cit-PtNPs, 0.2 ppm) and citrate-stabilized CeO_2_ NPs (cit-CeO_2_ NPs, 1000 ppm) were exposed to H_2_O_2_ (500 mM). The observed O_2_ % induced by the CAT-like activity of PtNPs reached 70%, while that induced by CeO_2_ NPs (5000-fold more concentrated) was 30% after 100 min. (D, E) Effect of PtNPs on the recovery of chemically induced ROS and apoptosis in primary rat cortex neurons. Treatments with 1 mM H_2_O_2_ for 5 min (D) and 5 μM antimycin A for 24 h (E) were used to induce ROS in the culture. ROS values were measured by using appropriate fluorescent probes (H_2_DCF and DHE, respectively). Apoptosis was measured through caspase 3/7 activity. Results are reported as means ± sem from *n* = 3 independent experiments and are normalized over untreated controls. **p < 0.01, ***p < 0.001, ****p < 0.0001; one-way ANOVA/Tukey’s tests. In all cases, neurons pretreated with 50 μg/mL RSA-coated PtNPs for 48 h showed a significant recovery of ROS amounts and apoptosis. (F) Representative TEM image of RSA-coated PtNPs internalized in primary rat cortex neurons (scale bar, 1 μm). Two representative intracellular vesicles containing PtNPs are indicated with numbers and magnified. Scale bars of magnified vesicles are 200 nm. N denotes the nucleus. (G) Estimated NP uptake quantification per cell from ICP-MS measurements. The uptake of RSA-coated PtNPs is significantly higher than that of citrate-stabilized PtNPs.

For this study, we chose PtNPs by virtue of their intrinsic enzyme-like activity with no need for surface shell engineering.^38^ The use of 4 nm sized PtNPs allows for maximizing the surface-to-volume ratio (increasing the surface atom number available for reaction) without losing the size and shape control of the nanostructures. Citrate-capped PtNPs were prepared as previously reported,^51^ yielding a reproducible monodisperse product with a narrow size distribution, as shown by transmission electron microscopy (TEM; Figure 1A) and dynamic light scattering (DLS; Figure S1).

Considering that albumin is the most abundant protein of the vitreous humor, PtNPs were coated with rat serum albumin (RSA) to obtain a biomimetic cloak and good colloidal stability in the biofluids of interest.^51^ This step also allowed us to eliminate excess citrate from the solution, minimizing osmotic and pH changes in the biofluid composition after the *in vivo* injection. Gel-shift assay analysis of RSA-coated PtNPs showed a sharp band characterized by slower electrophoretic mobility compared to pristine PtNPs, due to the larger size and lower charge of the complex (Figure 1B). The resulting PtNP-RSA complex formed by RSA adsorption greatly stabilized PtNPs in the electrolytic extracellular medium, emulating the effect of a protein corona. The antioxidant enzyme-like properties of these nanozymes were already studied in detail elsewhere.^49^ Here, we benchmarked the CAT-like properties of PtNPs with respect to the “gold standard” nanoceria (CeO_2_NPs), which are among the most investigated nanozymes for biological applications, particularly for retina protection.^33,41−47,56−60^ Using an oxygen sensor, we compared the oxygen developed by the CAT-like reaction over time (see Methods). For this experiment, the NP core size and surface chemistry were normalized and tested at lysosomal pH (pH 5). We found that PtNPs largely outperformed CeO_2_NPs also when the latter were employed in a 5000-fold excess (Figure 1C). Even if citrate was reported to favor the activity and colloidal stability of CeO_2_NPs, differences might be expected when using different ligands or more monodisperse samples (CeO_2_NPs tend to cluster in larger nanoassemblies). Nevertheless, we believe that the observed 3 orders of magnitude difference is a strong indication of the net superior activity of PtNPs.

We previously reported that biologically effective concentrations of PtNPs are fully biocompatible with several cell types, including mouse primary neurons, and do not virtually affect neuronal viability.^51^ Here, as an *in vitro* proxy for the *in vivo* study, we addressed the ability of our Pt-based nanoformulations to protect primary rat cortical neurons subjected to oxidative insults by either hydrogen peroxide or antimycin A which induces mitochondria ROS production, a process often involved in inflammatory responses.^61,62^ Neuronal cultures were evaluated for ROS levels using the fluorescent probes H_2_DCF and DHE and for the induction of apoptosis by determining caspase 3/7 activity (Figure 1D,E). In both assays, the preventive treatment of neurons with PtNPs significantly decreased the intracellular ROS levels and reversed ROS-induced apoptosis, testifying to their powerful activity against cell death caused by oxidative stress.

Using transmission electron microscopy (TEM) on primary rat cortical neurons treated with PtNPs, we confirmed the active internalization of the nanozymes that, as previously shown in a mouse model, were found within intracellular vesicles, consistent with their endocytosis and endolysosomal fate (Figure 1F).^51^ A quantification of the PtNP cellular uptake was also performed by mass spectrometry, confirming the key role of the protein coating in effective internalization. In fact, pristine PtNPs were only poorly internalized compared to RSA-stabilized PtNPs, likely due to their lower colloidal stability in biological media (Figure 1G).

### Light Damage Induces a Hotspot of Photoreceptor Degeneration in the Dorsal Retina of the Albino Rat

**Figure 2:**
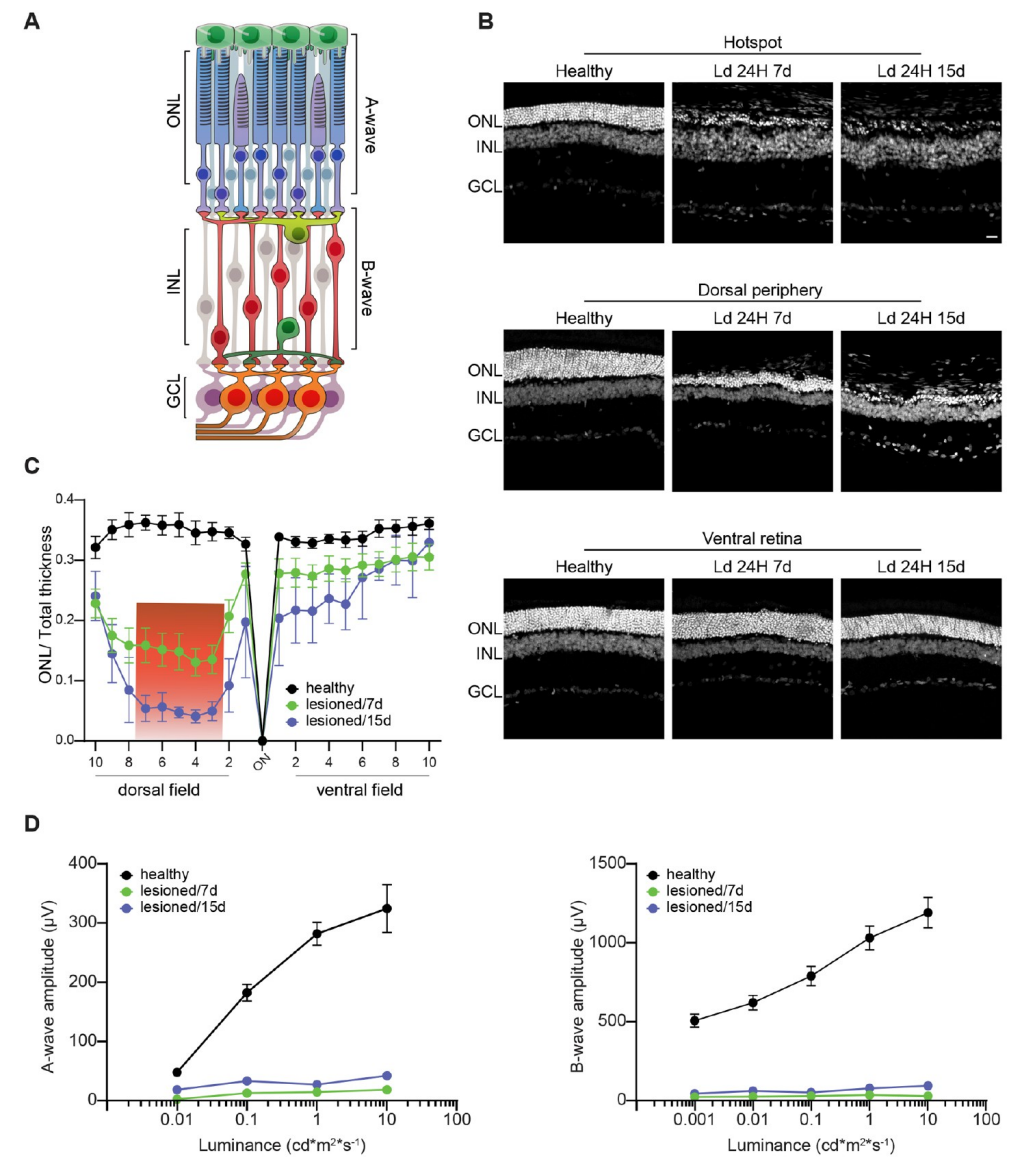
Morphofunctional characterization of the light-damage model of atrophic macular degeneration. (A) Schematic representation of the retinal circuitry and of the neuronal components responsible for the generation of the flash-electroretinogram (fERG) responses. The A-wave reflects the photoactivation of the PRs following a flash, while the B-wave is correlated to the propagation of the electrical stimuli from PRs to second-order neurons. (B) Morphological alterations and retinal function impairment assessed in albino SD rats reared in dim light (5–10 Lux) at two recovery times (7 and 15 days) after the light-induced retina lesion (24 h of light exposure to 1000 lux). Representative images labeled with bisbenzimide (white) of the hotspot in the dorsal retina (upper row), dorsal periphery (middle row), and ventral retina (lower row) of healthy rats and of rats that had been light-damaged for 24 h and analyzed 7 days (Ld 24H 7d) and 15 days (Ld 24H 15d) after the lesion. Of note, the presence of *rosettes* in Ld 24H 15d and photoreceptor loss were found in both lesioned groups, while no major changes were detected in the ventral retina. (C) The ONL thickness, normalized to the total retinal thickness (means ± sem), was calculated at 20 equidistant retinal positions from the dorsal periphery to the ventral periphery passing through the optic nerve (ON). The light damage causes a strong thinning of the ONL in the dorsal central retina (hotspot), where PRs are irreversibly damaged, surrounded by a penumbra area where degeneration can progressively spread (red box). Sample size: Healthy, *n* = 6; Ld 24H 7d, *n* = 6; Ld 24H 15d, *n* = 5. (D) Amplitudes (means ± sem) of the A-wave (left) and the B-wave (right) are plotted on a semilogarithmic scale as a function of the stimulus intensity (ranging from 0.001 to 10 cd m^2^ s^–1^). The exposure at 1000 lux for 24 h strongly reduces both fERG components in the lesioned animals. Sample size: Healthy, *n* = 8; Ld 24H 7d, *n* = 4; Ld 24H 15d, *n* = 4. Abbreviations: ONL, outer nuclear layer; INL, inner nuclear layer; GCL, ganglion cell layer. Scale bar: 20 μm.

To investigate whether PtNPs can prevent or mitigate the excessive oxidative stress and inflammation in the retina, we induced retinal photodamage in the albino rat, a well-established animal model to study PR degeneration in rodents that relies on the innate susceptibility to light of albino rats raised under dim-light conditions.^54,55,62−64^ The main feature of this model is that the PR damage starts and concentrates in a localized area of the dorsal retina called the “*hotspot*”. Photoreceptors in the hotspot fully degenerate during exposure to light, and degeneration spreads to the peripheral dorsal retina in the following days, leaving the ventral part almost intact, as shown by the thickness of the outer nuclear layer (ONL) analyzed 7 and 15 days after acute light damage (Figure 2A,B). In fact, the ratio between ONL thickness and total retina thickness progressively dropped over time only in the more light-sensitive dorsal retina, with the minimum occurring at the hotspot. Notwithstanding the same light exposure, no changes in ONL thickness were observed in the ventral retina due to its intrinsic resistance to light damage (Figure 2C).

We next characterized the functional impairment of the retina by recording the retinal electrical responses to brief light stimuli in dark-adapted animals (scotopic conditions) using flash electroretinography (fERG). We quantified the early “A-wave” hyperpolarizing response, directly linked to PR activation, followed by the “B-wave” depolarizing response due to postsynaptic activation of second-order retinal neurons. The A-wave (Figure 2D, left panel) and B-wave (Figure 2D, right panel) amplitudes recorded in healthy control littermates (black traces) were strongly impaired after 7 (green traces) and 15 (blue traces) days after light damage, matching the decrease in ONL thickness.

### Pretreatment with Platinum Nanozymes Attenuates Light-Induced Functional Damage to the Outer Retina

From a biological perspective, sub 50 nm particles are expected to permeate the vitreoretinal interface and the inner limiting membrane more efficiently while NPs with size >100 nm fail to permeate into the retina.^65^ To assess whether intravitreally injected PtNPs could spread through the retina and reach PRs, we performed TEM on ultrathin retina sections from *in vivo* PtNP-injected eyes. Interestingly, PtNPs were identified within PRs, both in the cell body and at the level of the outer segments (Figure S2).

**Figure 3:**
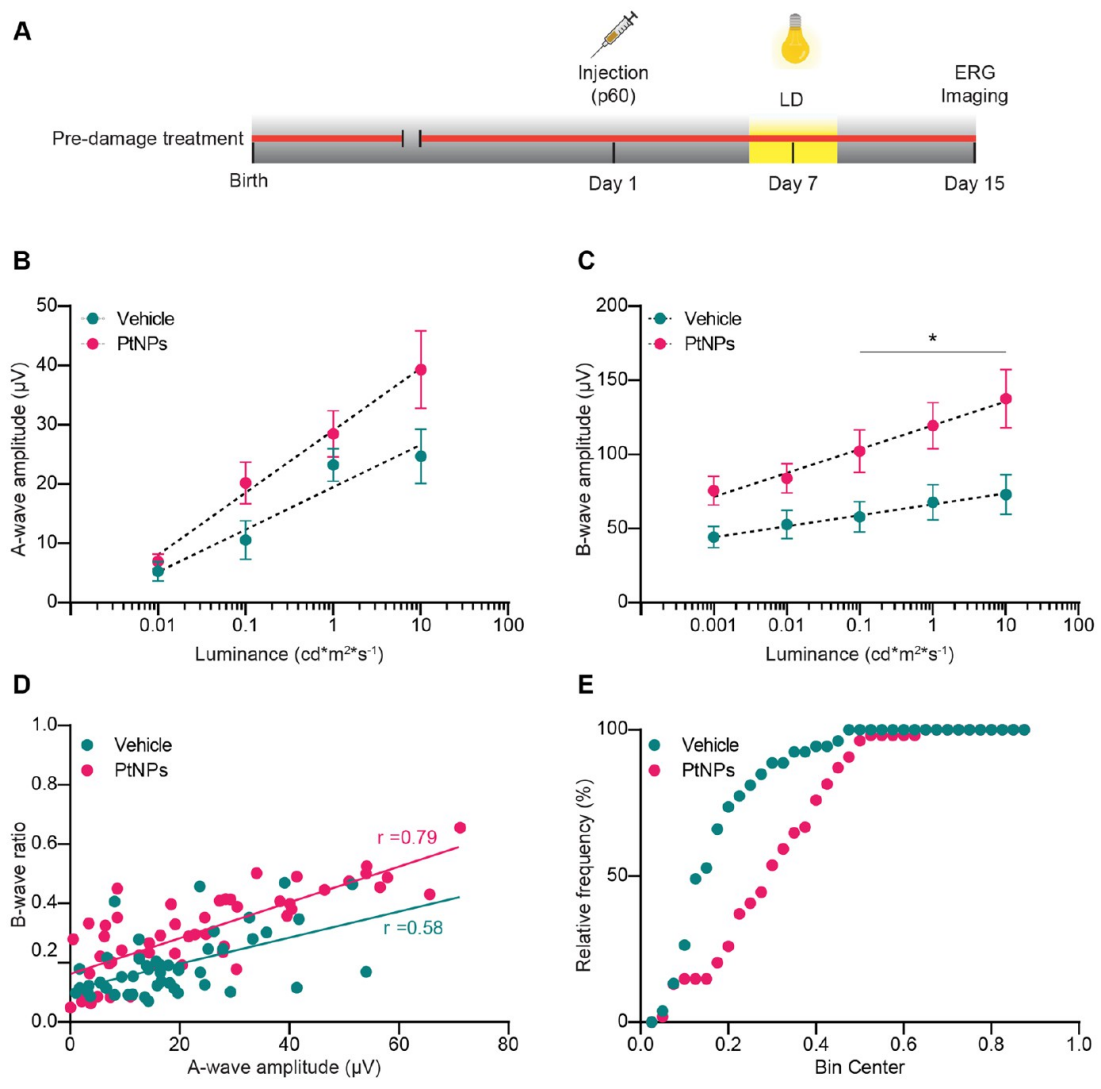
Effects of the preventive treatment with PtNPs on the electrical activity of light-damaged retinas. (A) Timeline of the experiments. Two-month-old albino SD rats reared in dim light were intravitreally injected with 2 μL of either PtNPs or vehicle (RSA). One week later, the animals were subjected to photo-oxidative damage by exposure to 1000 lux for 24 h. Fifteen days after the injection, fERG recordings were performed, and the retinal tissues were collected for morphological analyses (Imaging). (B, C) The amplitudes (means ± sem) of the A-wave (B) and the B-wave (C) are plotted on a semilogarithmic scale as a function of the stimulus intensity (ranging from 0.001 to 10 cd m^2^ s^–1^) for animals injected with either vehicle (blue) or PtNPs (red) before the light damage. Lesioned animals injected with PtNPs perform better than vehicle-injected animals, particularly for the B-wave at higher luminances, indicating an effect of PtNPs at the level of the photoreceptor/second-order neuron synapses. Sample size: *n* = 14 for both vehicle and PtNPs. *p < 0.05; two-way mixed ANOVA/Holm-Šídák’s multiple comparisons tests. (D) Correlation between the B-wave ratio (i.e., the amplitude of the experimentally recorded B-wave normalized by the healthy B-wave amplitude deduced from the A-wave amplitude) and the A-wave amplitude in lesioned animals injected with either vehicle (blue) or PtNPs (red) before light damage. The Pearson’s correlation coefficients (*r*) of the linear regression line are shown in the plot. (E) Cumulative distribution of the B/A wave ratios plotted in (D). *p* < 0.0001, Kolmogorov–Smirnov test.

Given the presence of PtNPs in PRs, we first analyzed whether they were active in preventing oxidative damage induced by light. In the preventive protocol (Figure 3A), 2-month-old dim-light-reared albino Sprague–Dawley rats were injected intravitreally with either vehicle (RSA) or a colloidal suspension of RSA-coated PtNPs 7 days before the induction of the light damage and subjected to ERG and immunohistochemical analysis 7 days later. Although ERG signals were still strongly reduced 7 days after light damage, we observed an improved functional recovery in the PtNP-treated retinas, testified by increased A-wave and B-wave amplitudes in response to full-field luminance stimuli. The 2-fold recovery of the B-wave was more prominent than that of the A-wave and was statistically significant (Figure 3B,C).

To deduce the signal transmission efficacy between PRs and second-order neurons, we calculated the ratio between the B-wave and A-wave amplitudes (Figure S3 and Table S1).^66^ Exclusive damage of PRs would lead to a reduced A-wave amplitude associated with a preserved B/A wave ratio. Instead, if synaptic transmission between PRs and bipolar cells is malfunctioning, then the B/A wave ratio would be degraded in the presence of a physiological A-wave amplitude. Thus, we plotted the normalized B-wave ratio for the different experimental groups, obtained by dividing the recorded B-wave amplitude by the expected B-wave amplitude that would be generated by the same A-wave in a healthy animal versus the A-wave values (Figure 3D; see Methods). It can be noted that the data relative to PtNP-injected animals (red) are distributed in the upper part of the graph compared to those of the animals injected with vehicle (blue), particularly for large A-wave amplitudes. Moreover, the cumulative distributions of the normalized B/A wave amplitude ratios demonstrate a significant difference between the two data populations (*p* < 0.0001; Kolmogorov–Smirnov test), confirming that the preventive treatment with PtNP had a more pronounced effect in the preservation of synaptic transmission between PRs and bipolar cells, rather than in the recovery of the physiological properties and survival of PRs (Figure 3E).

### Pretreatment with Platinum Nanozymes Attenuates the Astrocytic Reaction to Light Damage

**Figure 4:**
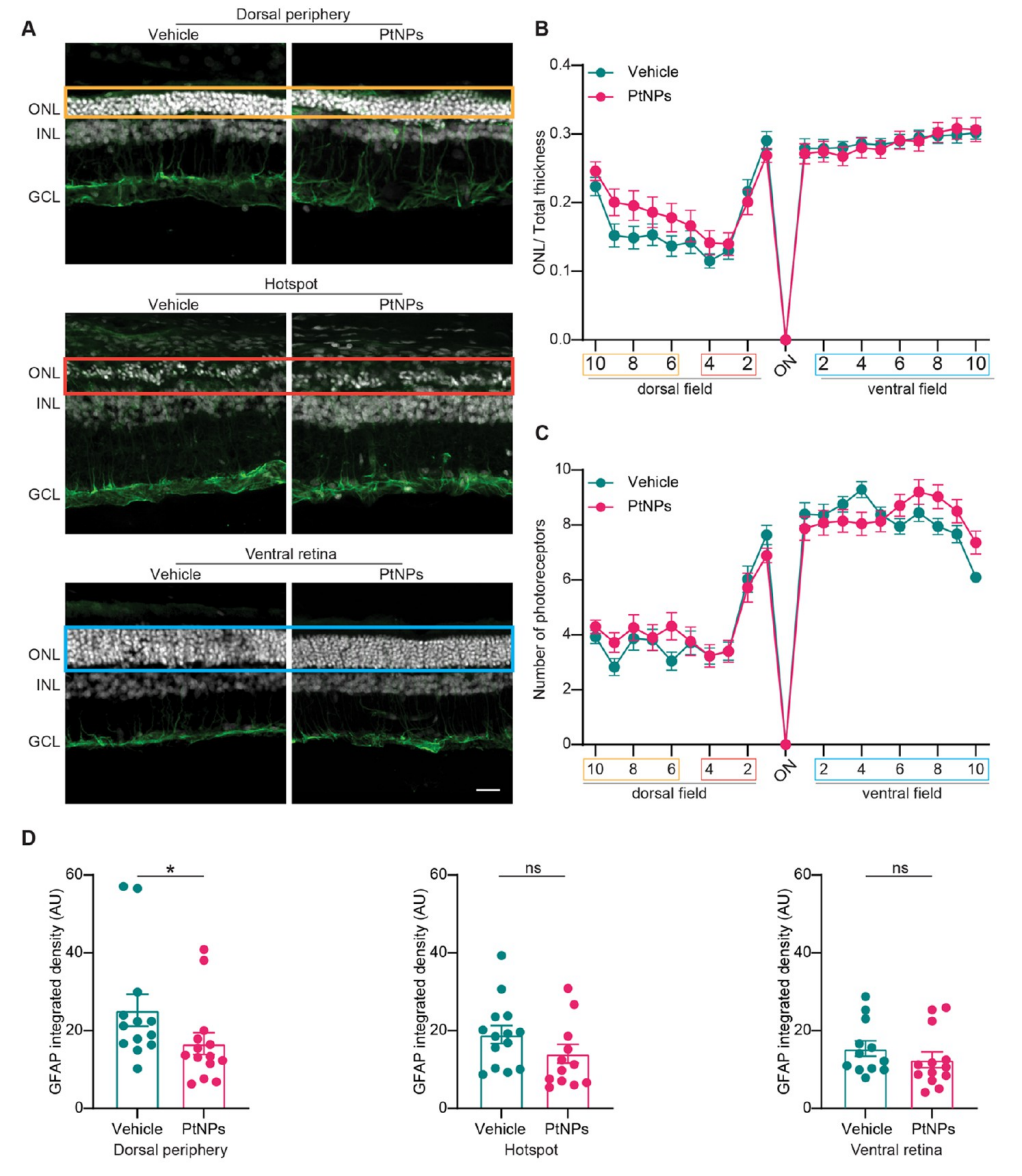
Effects of the preventive treatment with PtNPs on the morphology of light-damaged retinas. (A) Representative images of the dorsal periphery, hotspot, and ventral retina, labeled with bisbenzimide for nuclear labeling (white) and immunostained for the Müller cell marker GFAP (green). The images show only a slight effect on the ONL thickness in the dorsal retina in PtNP-pretreated animals compared with the vehicle-treated littermates. Abbreviations: ONL, outer nuclear layer; INL, inner nuclear layer; GCL, ganglion cell layer. Scale bar: 20 μm. (B, C) The ONL thickness normalized to the total retinal thickness (B) and the number of photoreceptor nuclear rows (C) are plotted at 20 equidistant retinal positions from the dorsal periphery to the ventral periphery passing through the optic nerve (ON) for animals injected with either vehicle (blue) or PtNPs (red) before light damage. Data are expressed as means ± sem. Colored boxes represent the corresponding analyzed areas shown in (A). Sample size: *n* = 14 for both vehicle and PtNPs. Two-way mixed ANOVA/Fisher’s LSD test. (D) Quantitative analysis of the integrated density of GFAP expression in the dorsal periphery (left; *n* = 14 and 12 for vehicle and PtNPs, respectively), hotspot (middle; *n* = 13 for both experimental groups), and ventral retina (right; *n* = 12 and 13 for vehicle and PtNPs, respectively). Bar plots represent the means ± sem with superimposed individual experimental points. PtNPs significantly reduce the GFAP expression in the dorsal periphery. ns, *p* > 0.05; *p < 0.05; Mann–Whitney *U*-test/unpaired Student *t*-test.

After the functional analysis, we investigated the preventive effects of PtNPs on retinal morphology and extent of the inflammatory response. The morphological assessment of the thickness of retinal layers (Figure 4A) showed that the ONL/total retina thickness ratio, a measurement of PR preservation after photodamage (Figure 4B), and the number of PRs in the ONL (Figure 4C) were not significantly different between vehicle- and PtNP-treated groups along the entire retina profile. While the ventral retina was not affected, the sharp light-induced decrease of ONL thickness in the dorsal retina was only slightly attenuated by the preventive administration of PtNPs, confirming the trend for an increase of the A-wave amplitude observed in Figure 3B. Then, the proinflammatory response of Müller cells/astrocytes and microglia to the light damage was evaluated by immunohistochemistry of the specific markers glial fibrillary acidic protein (GFAP) and ionized calcium-binding adapter molecule-1 (IBA1), respectively. While under physiological conditions GFAP is expressed in Müller cells at the level of the inner limiting membrane, upon stress induction, it gets overexpressed throughout the entire processes of the Müller cells. Pretreatment with PtNPs significantly reduced the integrated immunofluorescence density of GFAP in the periphery of the dorsal retina, but not in the hotspot, while no effect was observed in the ventral retina (Figure 4D). However, it did not affect the recruitment of microglia in the light-damaged area, as deduced from the number and distribution of IBA1-positive cells in vehicle- and PtNP-treated retinas (Figure S4).

Taken together, the data indicate that the preventive intravitreal injection of PtNPs can preserve synaptic transmission between PRs and second-order retinal neurons and reduce astroglial activation after intense photo-oxidative stress, suggesting that their action is mainly exerted on the inflammatory response that in turn affects retina signaling.

### Administration of Platinum Nanozymes after Light Damage Rescues the Electrophysiologic Activity of the Retina

**Figure 5:**
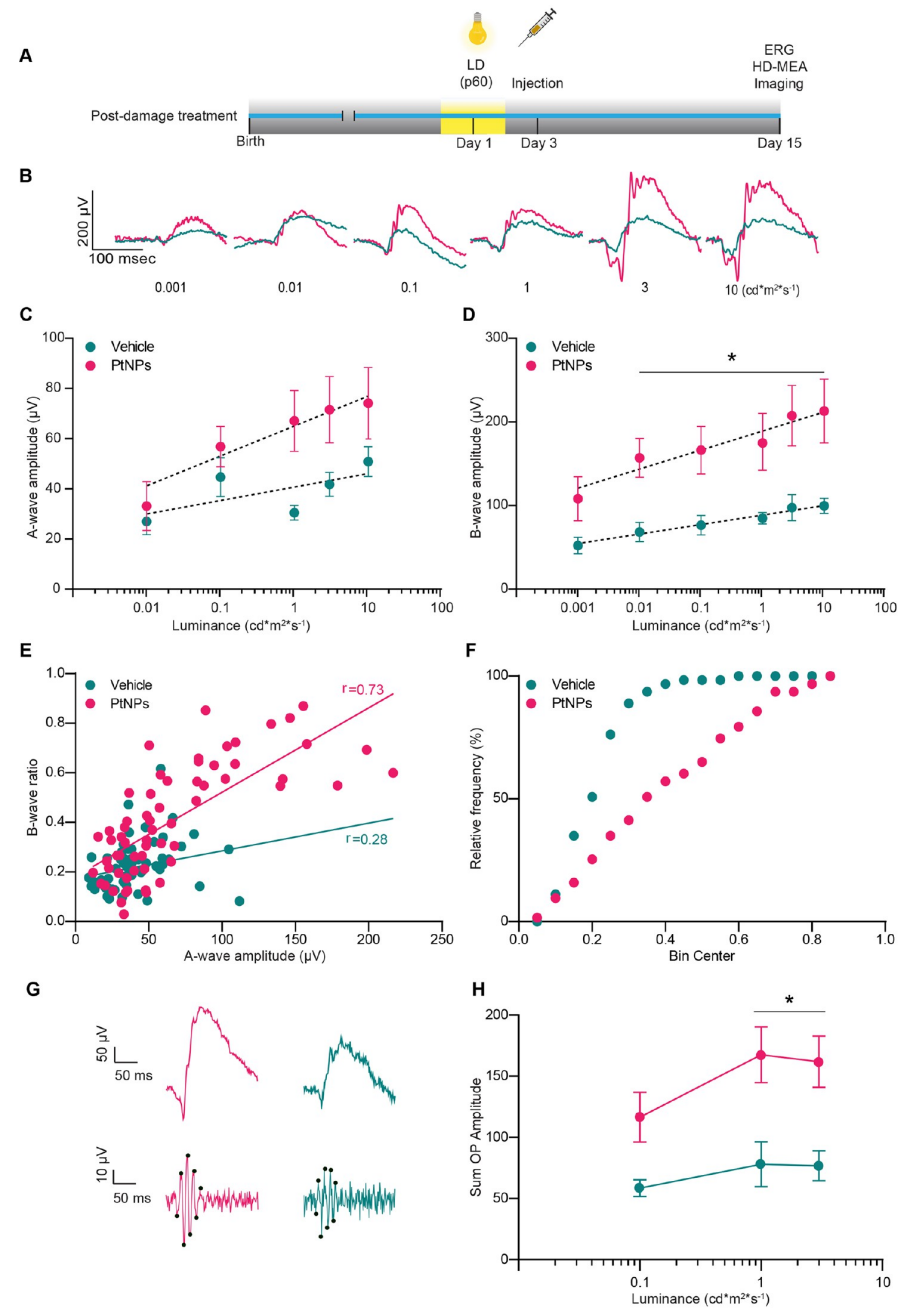
Effects of the postlesional treatment with PtNPs on the electrical activity of light-damaged retinas. (A) Timeline of the experiments. Two-month-old albino SD rats reared in dim light were subjected to photo-oxidative damage by exposure to 1000 lux for 24 h and, 48 h later, intravitreally injected with 2 μL of either PtNPs or vehicle (RSA). Fifteen days after the light damage, fERG recordings were performed, and the retinal tissues were collected for *ex vivo* electrophysiological (HD-MEA) and morphological analyses (Imaging). (B) Representative fERG traces evoked by light flashes of increasing luminance highlight the difference in the two groups for all tested luminances in animals injected with either vehicle (blue) or PtNPs (red) 24 h after light damage. (C, D) The amplitudes (means ± sem) of the A-wave (C) and the B-wave (D) are plotted on a semilogarithmic scale as a function of the stimulus intensity (ranging from 0.001 to 10 cd*m^2^*s^–1^) for animals injected with either vehicle (blue) or PtNPs (red) 24 h after light damage. At all tested luminances, the animals injected with PtNPs show larger amplitudes than vehicle-injected animals, particularly for the B-wave, suggesting a positive effect of PtNPs in preserving retinal function, even after severe light damage. **E.** Correlation between the B-wave ratio (i.e., the amplitude of the experimentally recorded B-wave normalized by the healthy B-wave amplitude deduced from the A-wave amplitude) and the A-wave amplitude in lesioned animals injected with either vehicle (blue) or PtNPs (red) after the light damage. The Pearson’s correlation coefficients (*r*) of the linear regression line are shown in the plot. (F) Cumulative distribution of the B/A wave ratios plotted in (D). The difference between the two experimental groups suggests the ability of PtNPs to preserve both an efficient synaptic transmission between PRs and second-order neurons and an enhanced PRs activation. *p* < 0.0001, Kolmogorov–Smirnov test. (G, H) Representative recordings (G) and mean (±sem) sum of amplitudes (H) of oscillatory potentials as a function of luminance (0.1, 1, and 3 cd m^2^ s^–1^) in PtNP- and vehicle-treated animals. Peaks of OPs are labeled with black dots. Sample size: vehicle, *n* = 14; PtNPs, *n* = 16. *p < 0.05; two-way mixed ANOVA/Holm-Šídák’s tests (C, D, H).

We next analyzed whether PtNPs are also active to counteract the oxidative stress/inflammation/neuronal death cycle when administered after light damage (Figure 5A).

Two-month-old Sprague–Dawley rats reared in dim light were first subjected to the light damage, followed 24 h later by an intravitreal injection of either vehicle or PtNPs. We then left 2 weeks to recover from surgery, and on the last day, we performed *in vivo* fERG measurements and *ex vivo* electrophysiological and morphological analyses. Representative fERG recordings are shown in Figure 5B. Analysis of the average amplitude of the A-wave (Figure 5C) and the B-wave (Figure 5D) revealed that the postlesion treatment with PtNPs enhanced fERG responses with respect to vehicle-injected controls. The A-wave recovery was more prominent compared to that obtained in the preventive protocols (around 60 μV for luminances above 0.1 cd m^2^ s^–1^), compared to an average of 30 μV for the preventive protocol in the same luminance range (Figure 5C). Postlesion PtNPs significantly enhanced the B-wave that displayed a 2-fold increase in amplitude over the whole luminance range with respect to vehicle-injected controls (Figure 5D). While in healthy retinas (see Figure 2D), the luminance response curve of the B-wave follows the sigmoid-like Naka–Rushton function,^67^ in our light-damaged retinas it remained almost linear, irrespective of the treatment. Given the more significant effects recorded for the B-wave as compared to the A-wave, we investigated whether PtNPs could have improved visual information transfer from PRs to bipolar cells also in the postlesional treatment. When the ratio between the normalized B-wave ratios was plotted versus the respective A-wave amplitude, we found a much higher upper shift of the experimental points in the PtNP-treated group with respect to the vehicle-treated one (Figure 5E). Furthermore, the cumulative distributions of the normalized B/A wave amplitude ratios demonstrate a highly significant difference between the two data populations (*p* < 0.0001; Kolmogorov–Smirnov test; Figure 5F). We finally asked whether the stimulating effects on synaptic transmission by PtNPs observed in the outer retina also impact inner retinal transmission. To answer this question, we measured the amplitude of the oscillatory potentials (OPs), a series of wavelets overlapping the ascending phase of the B-wave and reflecting the interactions among bipolar cells, amacrine cells, and ganglion cells in the inner retina. We observed significantly larger OP amplitudes in the lesioned group treated with PtNPs with respect to the vehicle-treated one, consistent with a global reactivation of retinal processing (Figure 5G, H).

### Lesioned Retinas Treated *In Vivo* with Platinum Nanozymes after Light Damage Display a Selective Preservation of ON Retinal Ganglion Cell Responses

**Figure 6:**
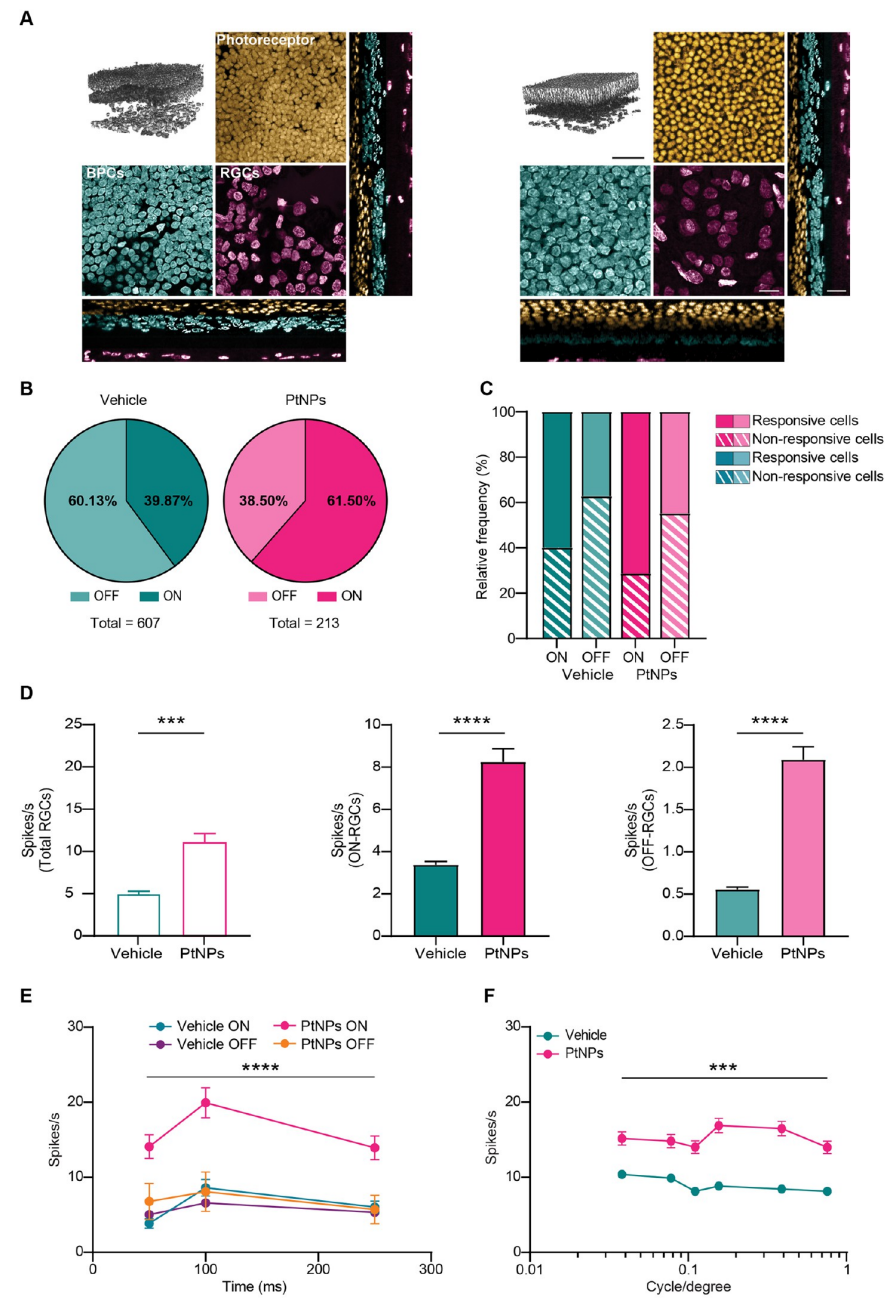
Effects of the postlesional treatment with PtNPs on retinal ganglion cell firing in the dorsal light-damaged retina. (A) Upper left panel: 3D reconstruction of representative dorsal hemiretinas from vehicle (left)- and PtNP (right)-treated rats 24 h after the light damage (scale bar, 50 μm). Other panels: *Z*-axis magnification of each retinal layer and the orthogonal *XZ*, *YZ* projections (scale bar: 20 μm). The PtNP treatment ameliorates the retinal architecture, which is notably altered in vehicle-treated retinas due to ongoing degeneration. (B) Pie charts showing the percentage of ON (dark color) and OFF (light color) RGCs on the total number of active cells recorded for each dorsal hemiretina in vehicle- (blue)- and PtNP (red)-treated rats 24 h after the light damage. Of note, there is a higher occurrence of active ON-RGCs in the PtNP-treated retinas (*p* < 0.0001, Fisher’s exact test). (C) Percentage of responsive cells for each RGC polarity. ON-RCGs are more responsive in both groups. Treatment with PtNPs further increases the percentage of responsive ON-RGCs, while no differences are observed for OFF-RGCs. (D) Bar plots (means ± sem) of the spiking activity of total RGCs (left; *n* = 607 and 213 cells for vehicle and PtNPs, respectively), ON-RGCs (middle; *n* = 242 and 131 cells for vehicle and PtNPs, respectively), and OFF-RGCs (right; *n* = 365 and 82 cells for vehicle and PtNPs, respectively) in response to a 250 ms full-field flash stimulation in dorsal hemiretinas from vehicle (blue)- and PtNP (red)-treated rats 24 h after the light damage. These graphs reveal an increment for the PtNP-treated retinas and display a generalized and significant increase in RGC firing activity. (E) Temporal dynamics of RGC firing evoked by full-field flash stimulation. While no difference is present for the OFF-RGCs, significantly higher spiking activity is evident for the ON-RGCs of the PtNP-treated, but not vehicle-treated, retinas. (F) RGC spiking activity evoked by reverting gratings with spatial frequency ranging from 0.2 to 0.8 cpd to assess spatial discrimination capabilities. The light-lesioned retina treated with PtNPs displays a significant increase in spatial resolution at all tested frequencies with respect to vehicle-treated retinas. **p < 0.01, ***p < 0.001, ****p < 0.0001; Mann–Whitney *U*-test (D), two-way mixed ANOVA/Holm-Šídák’s tests (E, PtNPs ON-RGCs versus vehicle ON-RGCs; F, PtNPs versus vehicle).

At the end of the *in vivo* recordings and before histochemical analyses, retinas were acutely explanted and positioned on HD-MEA chips in the epiretinal configuration for *ex vivo* electrophysiological analysis. Dorsal and ventral parts of each retina were divided and recorded separately. A 3D-reconstruction of recorded dorsal hemiretinas with close-ups at the level of ONL, INL, and RGC layers shows that the retinal architecture of the vehicle-treated group was severely altered, while that of the PtNP-treated group was substantially preserved (Figure 6A).

We then challenged the hemiretinas using two sets of stimuli: (i) white (ON) or black (OFF) full-field stimulation (lasting 50, 100, and 250 ms at 0.25 Hz) to study polarity and temporal dynamics of RGC responses; (ii) squared luminance gratings alternating at 1 Hz and with spatial frequency ranging from 0.03 to 0.80 cycles/degree (cpd) to study spatial filtering and spatial resolution properties. Due to the individual variability and the slightly variable electrical coupling of the retinas with the chip, we recorded different numbers of RGCs from each sample totalizing 607 and 213 RGCs in the dorsal retinas and 984 and 193 RGCs in the ventral retinas for vehicle- and PtNP-injected groups, respectively, that were classified as ON or OFF as specified in Methods (Figure 6B and Figure S5B). In the dorsal retina, the ON-RGCs were the more protected cell population by PtNPs. In fact, the number of ON-RGCs in the PtNP-injected group was significantly higher than the number in the vehicle-treated group (39.8% and 61.5% of active cells for vehicle and PtNPs, respectively). The percentage of responsive RGCs for each stimulus polarity was then calculated by considering those RGCs firing ≤1 action potential during ON or OFF stimulation as “nonresponsive” and those firing >1 action potential as “responsive”. The analysis also showed that the treatment with PtNPs brought about an increase in responsive over nonresponsive RGCs in the dorsal retina, while no major differences were observed for the OFF-RGC population in the same area (Figure 6C).

When we analyzed the total RGC spiking response (left), as well as the individual spiking rates of ON (middle) and OFF (right) RGC populations (Figure 6D), significant increases in the spiking activity of all RGC populations in PtNP-treated retinas compared to those in vehicle-injected ones were observed. The analysis of the temporal dynamics of the response of ON- and OFF-RGCs in the dorsal retina confirmed that the ON-RGC population of the PtNP-treated group exhibited dynamics significantly higher than that of the vehicle-injected group, while the OFF-RGC population did not show any significant improvement following PtNP treatment (Figure 6E).

Finally, when the dorsal hemiretinas were stimulated with patterned stimuli at varying spatial frequencies, the evoked spiking activity of RGCs from the PtNP-treated group was significantly higher than that of the flat response curve of the vehicle-treated group (Figure 6F), confirming the observed increase in OPs (see Figure 5). By mimicking the *in vivo* retinal spatial discrimination, this *ex vivo* experiment shows that PtNP could preserve spatial organization of the RGC receptive fields, more efficiently encoding patterned stimuli. Moreover, the shape of the spatial resolution curve of the PtNP-injected group suggests the preservation of lateral inhibition in both long and short ranges, a phenomenon that is virtually absent in vehicle-treated retinas.

The corresponding results collected from the ventral part of the retina are summarized in Figure S5. The 3D reconstruction of the ventral retina layers did not exhibit overt architectural changes, irrespective of the treatment (Figure S5A). No significant differences were observed in the number of ON- and OFF-RGCs in vehicle- and PtNP-injected groups (45.33% and 48.19% of the active cells for vehicle and PtNPs, respectively; Figure S5B). The percentages of responsive and nonresponsive ON-RGCs (Figure S5C) and the total RGC spiking response (Figure S5D) also did not differ significantly between the two treatments. The analysis of the temporal dynamics of the response of ON- and OFF-RGCs in the ventral retina (Figure S5E) revealed a predominance of firing activity for the ON-RGCs over the OFF-RGCs that was similar in vehicle- and PtNP-treated rats. This result is in contrast with the respective recordings in the dorsal retina, where the ON-RGC population was the more sensitive population to both light damage and the protective effect of PtNPs.

### Administration of Platinum Nanozymes after Light Damage Markedly Attenuates Photoreceptor Degeneration and Retinal Inflammation

**Figure 7:**
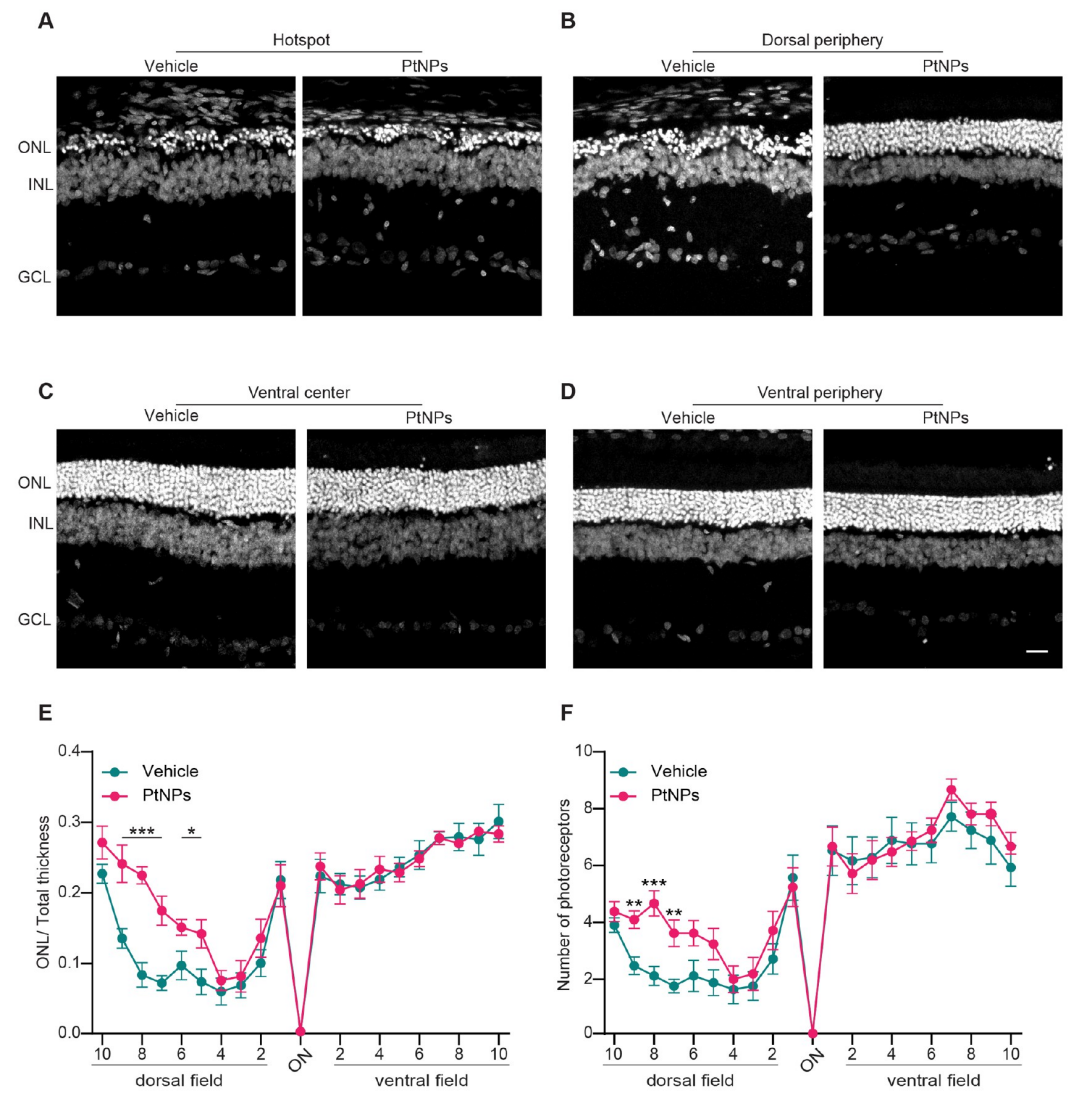
Effects of the postlesional treatment with PtNPs on the morphology of light-damaged retinas. (A–D) Representative cross sections of the dorsal periphery, hotspot, and ventral retina, labeled with bisbenzimide for nuclear labeling (white). Abbreviations: ONL, outer nuclear layer; INL, inner nuclear layer; GCL, ganglion cell layer. Scale bar: 20 μm. (E, F) The ONL thickness normalized to the total retinal thickness (E) and the number of photoreceptor nuclear rows (F) are plotted at 20 equidistant retinal positions from the dorsal periphery to the ventral periphery passing through the optic nerve (ON) for animals injected with either vehicle (blue) or PtNPs (red) 24 h after light damage. Data are expressed as means ± sem. Sample size: vehicle, *n* = 8; PtNPs, *n* = 10. *p < 0.05, **p < 0.01, ***p < 0.001; two-way mixed ANOVA/Fisher’s LSD tests. PtNPs significantly preserve both the ONL thickness and the number of PRs in the dorsal retina periphery, limiting the extension of the damaged area at the hotspot level only and preventing the spread of the degeneration to the penumbra area. The ventral area is not affected by light damage.

The effects of the postlesional treatment with PtNPs on retinal morphology were evaluated by nuclear staining with bisbenzimide and immunohistochemistry for the proinflammatory markers GFAP and IBA1. Opposite to what was observed in the preventive protocol, treatments of photodamaged retinas with PtNPs significantly preserved the number of PR nuclei in the ONL (Figure 7).

Although PtNPs failed to preserve the PR population in the hotspot (in which, however, better morphology preservation with fewer *rosettas* was observed; Figure 7A), the peripheral dorsal retina was significantly protected compared to the control group (Figure 7B). As shown in Figures 2 and 4, no difference between the two experimental groups was observed in the ventral part of the retina, which is more resistant to the photodamage and is not apparently affected morphologically (Figure 7C,D). The quantitative analysis of the ONL/total retinal thickness ratio (Figure 7E) and the number of PR rows (Figure 7F) as a function of the distance from the optic nerve confirms the highly significant preservation of the PRs population in the ONL of the peripheral dorsal area in the presence of PtNPs.

**Figure 8:**
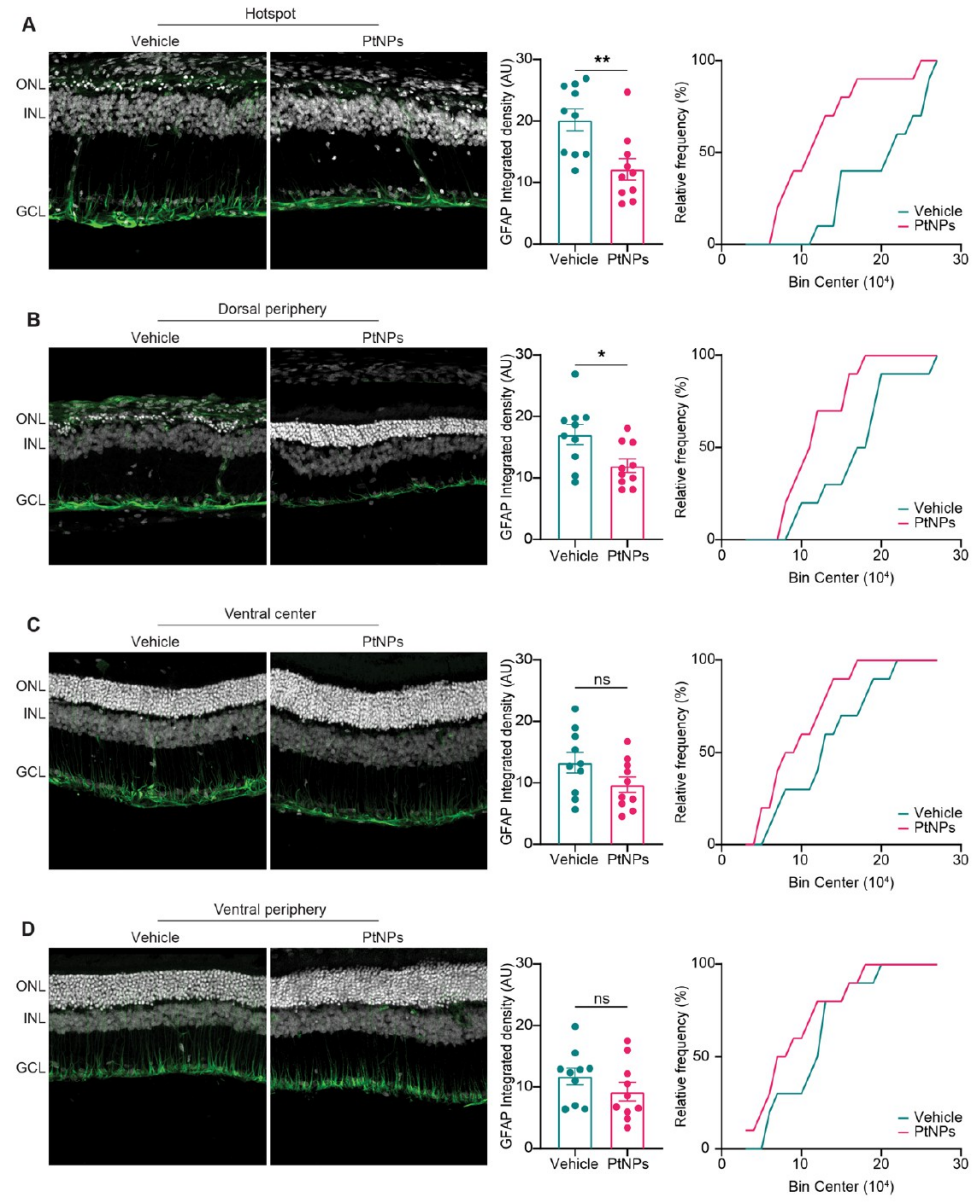
Effects of the postlesional treatment with PtNPs on astrocyte activation in light-damaged retinas. (A–D) Retinas from animals injected with either vehicle (blue) or PtNPs (red) 24 h after the light damage were analyzed for GFAP expression in the hotspot (A) and periphery (B) of the dorsal retina and in the center (C) and periphery (D) of the ventral retina. Left panels: representative retinal cross sections immunolabeled for the Müller cell marker GFAP (green) merged with bisbenzimide nuclear labeling (white). Abbreviations: ONL, outer nuclear layer; INL, inner nuclear layer; GCL, ganglion cell layer. Scale bar, 20 μm. Middle panels: quantitative analysis of the integrated density of GFAP expression. Bar plots represent the means ± sem with superimposed individual experimental points. PtNPs significantly reduce the extent of the GFAP expression in the dorsal retina. Sample size: vehicle, *n* = 10; PtNPs, *n* = 10. *p < 0.05, **p < 0.01; Mann–Whitney *U*-test/unpaired Student *t*-test. Right panels: corresponding cumulative frequency distribution curves (binning width: 10000). PtNP injection significantly reduced the upregulation of GFAP in the Müller cells both in the hotspot and in the adjacent dorsal periphery with respect to vehicle-treated retinas (A, *p* = 0.015; B, *p* = 0.078; Kolmogorov–Smirnov test). No differences are present between the two experimental groups in the ventral areas (C, *p* = 0.699; D, *p* = 0.699; Kolmogorov–Smirnov test).

We then analyzed the impact of the treatment on the inflammatory response triggered by photodamage by evaluating GFAP expression in Müller cells/astrocytes and the number and activation state of microglial cells. In agreement with the functional recovery, treatment with PtNPs markedly reduced GFAP expression not only in the dorsal peripheral area, as already observed for the preventive protocol (see Figure 4), but also in the hotspot, the most affected area by the photo-oxidative insult (Figure 8A,B, left). The sharp protective effect of PtNPs is displayed both by the quantitative analysis of the integrated density of GFAP expression (middle) and by the cumulative frequency distribution curves (right). As expected, the ventral retina remained almost unaffected by light damage under both experimental conditions (Figure 8C,D).

**Figure 9:**
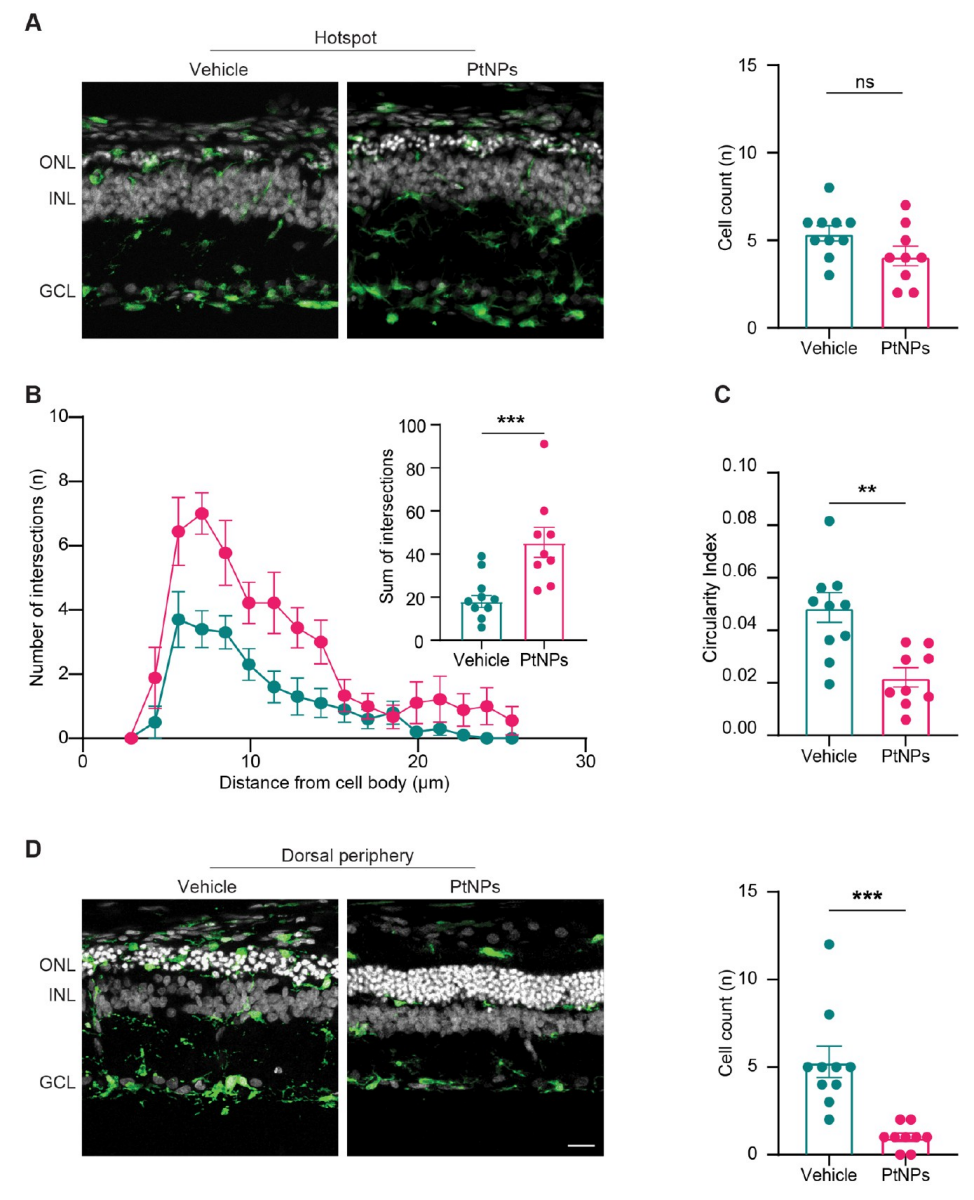
Effects of the postlesional treatment with PtNPs on the activation of microglia in light-damaged retinas. Retinas from animals injected with either vehicle (blue) or PtNPs (red) 24 h after the light damage were immunolabeled for the microglial marker IBA1 (green) merged with bisbenzimide nuclear labeling (white). Abbreviations: ONL, outer nuclear layer; INL, inner nuclear layer; GCL, ganglion cell layer. Scale bar: 20 μm. (A) Dorsal retina hotspot. Representative IBA1-stained cross sections (left) and bar plot of the mean (±sem) number of IBA1-positive microglial cells counted in the ONL with superimposed individual experimental points (right). (B) Sholl analysis showing the mean (±sem) number of intersections of microglial processes with shells drawn at increasing distances from the soma. Inset: bar plot showing the mean (±sem) sum of intersections calculated from the cell body to the maximal branch extension. (C) Circularity index of microglial cells in the ONL of the hotspot in the dorsal retina. The index was calculated using the Fiji software plugin. A circularity value of 1.0 indicates a perfect circle, while its decrease toward zero indicates an increasingly elongated polygon. Bar plots represent the means (±sem) with superimposed individual experimental points. The more elongated and polygonal-like shape observed in the PtNP group reveals decreased activation of the microglial cells. (D) Dorsal retina periphery. Representative IBA1-stained cross sections (left) and bar plot of the mean (±sem) number of IBA1-positive microglial cells counted in the ONL with superimposed individual experimental points (right). Microglial cells usually reside in the inner retina playing a surveillance role. After light damage and photoreceptor degeneration, microglial cells migrate to the outer retina, retracting pedicles and assuming a more amoeboid shape. PtNPs significantly reduce their infiltration in the ONL. Sample size: vehicle, *n* = 10; PtNPs, *n* = 9. **p < 0.01, ***p < 0.001, unpaired Student *t*-test (A–C), Mann–Whitney *U*-test (D).

We finally analyzed IBA1-positive microglial cells. Under physiological conditions, resident microglial cells are localized in the inner part of the retina and display a ramified shape. After stress induction, microglial cells lose their processes, assume an ameboid shape, and migrate to the outer retina, starting to recruit macrophages from the choroidal circulation. The number of microglial cells in the hotspot of the dorsal retina of PtNP-treated groups was not altered (Figure 9A). However, to address the microglial proinflammatory activation state, we computed the Sholl and circularity index analyses for each IBA1-positive cell. A Sholl analysis of hotspot microglial processes showed a 2-fold higher number of intersections in PtNP-treated retinas with respect to vehicle-treated retinas, indicating a significantly more ramified morphology (Figure 9B). This treatment-dependent change was confirmed by the circularity index analysis, which showed that microglial cells in PtNP-treated retinas maintained the low circularity index characteristic of quiescent, ramified microglia (Figure 9C), while microglia of vehicle-treated retinas assumed a circular shape. The anti-inflammatory effect of PtNPs was also evident in the dorsal periphery, where the number of microglial cells was markedly reduced by over 4-fold (Figure 9D), while no effects were observed in the ventral retina (Figure S6).

Taken together, the data reveal that the postlesional PtNP treatment is more effective than the preventive protocol in preserving PR number and in reducing Müller cell and microglial proinflammatory responses in the dorsal retina.

## Conclusions

Rare-earth NPs have been widely used for their antioxidant and anti-inflammatory potential in an attempt to alleviate oxidative stress in various neurodegenerative diseases. One of these diseases is atrophic AMD, a so far uncurable age-related macular degeneration in which long-term oxidative stress is one of the causative factors. Another condition in which oxidative damage can produce retinal degeneration is spaceflight-associated neuro-ocular syndrome attributable to sustained exposure to microgravity and cosmic radiation.^68^ In particular, high doses of nanoceria were repeatedly shown to prevent light-induced oxidative damage to the most light-sensitive retinal area, i.e., the dorsal retina that is generally not adapted to direct irradiation, after intravitreal or topical administration.^41−47,56−60^ PtNPs were recently characterized for their intrinsic multiple enzyme-like properties and their high biocompatibility and stability in biological media make them a very versatile tool to potentiate and replace the endogenous antioxidant activity of intracellular systems.^38,49^ Depending on the protein concentration in biological media, PtNPs are coated with protein corona that shields the catalytic activities of the PtNPs in the extracellular environment.^51^ However, after cellular internalization and lysosomal accumulation, the antioxidant enzyme-like properties of PtNPs are completely recovered and boosted thanks to the proteolytic environment (corona degradation) and the acidic pH (enhancing PtNP performances).^51^ Here we confirmed these properties and intracellular fate in primary rat neurons, showing that PtNPs protect neurons from ROS generation and inflammation-induced apoptosis. Moreover, thanks to their very small size (4 nm) and the RSA coating, we demonstrated their ability to cross the inner limiting membrane and permeate the entire retina thickness, reaching PRs.

As was done in previous papers using rare-earth NPs, we assessed the neuroprotective effects of PtNPs in an induced PR degeneration model caused by photo-oxidative damage in dim-light-reared albino rats. The light-damaged albino rat model is interesting in several respects. First, it is a model induced in healthy adult animals, reducing the possible compensatory effects that may affect genetic-based models. The model is a surrogate of AMD in humans, as it has a nongenetic origin and acutely reproduces the chronic oxidative stress that PRs undergo during life. It is characterized by a confined, but progressive, PR degeneration that mimics the progressive perifoveal rod/foveal cone death occurring in human atrophic AMD in a fovealess animal model. Third, as the PR photodamage is induced by the accumulation of oxidative stress, it is a particularly suitable model to assess the efficacy of antioxidant treatments, such as PtNPs. While photo-oxidative damage directly and irreversibly triggers PR death in the hotspot, oxidative stress and the ensuing chronic inflammatory response cause the progression of PR death throughout the dorsal peripheral retina.^2,3,54,55,63,64,69,70^ Thus, while dead PRs in the hotspot are hardly recoverable, PRs of the peripheral dorsal area are not irreversibly damaged and are the ideal target for therapy.

We administered RSA-coated PtNPs to the eye intravitreally, a widely used administration route in humans, in low doses and according to two strategies: (i) preventive protection in which PtNPs were administered 1 week before the light damage and (ii) postlesional therapy in which PtNPs were injected 1 day after the photolesion. In contrast with previous works, which used high concentrations of nanoceria, the preventive protocol was unable to effectively counteract PR death at the tested concentration but significantly decreased the light lesion-induced astrogliosis and increased the B-wave of the ERG, testifying to an improvement of the activation of second-order retinal neurons by the PRs. On the other hand, administration of PtNPs 24 h after the photolesion (curative protocol), a better strategy to use NPs on a diagnosed disease, had a stronger effect. PtNPs significantly preserved both the ONL thickness and the number of PRs in the dorsal retina periphery, limiting the extension of the damaged area at the hotspot level only and preventing the spread of the degeneration to the penumbra area. This effect was particularly evident by recording large populations (hundreds) of RGCs in the explanted retina 2 weeks after the photodamage. PtNPs greatly increased the number of active ON-RGCs in the dorsal retina, improving their firing activity in response to light stimuli and enhancing the spatial resolution in response to patterned stimuli at various spatial frequencies. The latter effect is consistent with the increased signaling between PRs and second-order neurons that preserves the functionality of lateral circuits essential for contrast and spatial sensitivity (1 cpd in the rat corresponds to approximately 60 μm at the retinal level).

Although effective, treatments with antioxidant PtNPs cannot regenerate apoptotic PRs. Thus, the main effect is that of slowing degeneration and rescuing PRs that are not irreversibly damaged and located in the penumbra area adjacent to the hotspot (peripheral dorsal retina). In this respect, an important effect is the potent downregulation of astrogliosis and microgliosis in the dorsal retina, which lowers the extent of retinal inflammation and contributes to functional recovery. The expression of the Müller cell/astrocyte marker GFAP was markedly downregulated in the whole dorsal retina, and microglial cells that are potently activated in the hotspot were returned to a quiescent state. Although these effects can be in part secondary to the decreased extent of degeneration, they likely depend on the direct anti-inflammatory activity of PtNPs. ROS are key signaling molecules primarily involved in the regulation and progression of inflammatory disorders.^71^ In this respect, the downscaling effect of PtNPs on microglial activation could be relevant, as it was recently reported that the microglia–neuronal axis can induce autophagy dysfunction in neurons through the secretion of chemokines activating the neuronal mTORC pathway.^72,73^

Several factors can contribute to the higher efficacy of the postlesional treatment with PtNPs. In both the preventive and curative protocols, the time interval between the intraocular PtNP injection and the physiological analysis was constant (15 days), while the main difference between the two protocols is the absence or presence of light-induced inflammation at the time of PtNP injection. While in the preventive protocol, PtNPs only “strengthened” the antioxidant capacity of the normal retina before the light damage, in the postlesional protocol, they came into play at the peak of retina inflammation due to light damage. The concomitant immune system activation favoring the PtNP uptake at the inner limiting membrane level would potentiate the anti-inflammatory effect. Indeed, PtNPs exhibit their maximum catalytic effects at low pH, most likely in the lysosomal compartment^51^ that is particularly active during inflammation in microglial cells, and microglial targeting has been recently reported for nanozyme-based treatment of neurodegenerative diseases.^52^ The data suggest that the primary effect of PtNPs consists of reducing the vicious cycle of the inflammatory response rather than preventing the photo-oxidative damage caused by light, being more effective when applied to tissue already affected by an inflammatory state. Indeed, PtNPs administered postlesionally reduced both the astroglial and microglial inflammatory reaction, while preventively administered PtNPs only slightly attenuated astrocytic reaction to light damage. We cannot exclude, however, that the PtNP concentration in the preventive protocol might have decreased at the time of the light damage or that the shorter time window between the light damage and the physiological assessment might have contributed to the lower efficacy of the preventive treatment.

Bio- and immune-compatibility tests performed so far on PtNPs are encouraging, as they have high chemical stability in the biological/cellular environment (unlike, for instance, AgNPs).^38,49,51,74^ PtNPs are already commercially available in some countries (such as Japan) as food supplements, skin care creams, and other cosmetic products. Nevertheless, general concerns and risks related to the use of nanomaterials in medical treatments of ocular diseases should be considered, as PtNP nanoformulations are not yet clinically approved. It should be acknowledged that given the superior catalytic performances of PtNPs, therapeutic effects can be obtained at very low doses, thus limiting adverse effects. Regarding the PtNP administration route, the risk of intravitreal injections is relatively low, as they are already widely used in the therapy of wet macular degeneration with anti-VEGF monoclonal antibodies with an estimated 6 million treatments annually in the USA^75^ and a complication rate of 1.9% of all injections.^76^ Moreover, the risk of intraocular injections could be further minimized in the future by developing appropriate eyedrop formulations for topical administration, as recently reported for other NPs.^59^

In conclusion, the data indicate that the intravitreal injection of PtNPs can preserve retinal physiology after intense photo-oxidative stress. Visual information processing was improved by preserving synaptic transmission between PRs and second-order retinal neurons and reducing glial activation. This suggests that the PtNP action is mainly exerted on the inflammatory response to the photo-oxidative damage, known to be one of the main events in the progression of retina degeneration. In turn, PtNP activity affects retina signaling, suggesting that this treatment can be effective for most retinal degenerative processes and as a prevention for high-risk retinal insults by electromagnetic radiation (such as in spacecraft journeys). It is tempting to speculate that PtNPs can effectively break the vicious cycle linking ROS, degeneration, and inflammation by exerting potent combined antioxidant and anti-inflammatory actions. Although further studies on more reliable models of atrophic AMD, such as the sodium iodate lesioned pig retina,^77^ are needed, the increased PR survival, decreased inflammation, and improved visual performances in degenerated retinas make PtNPs a potential strategy to cure AMD.

## Methods

### Ethical Approval and Animal Handling

Albino Sprague–Dawley (SD) pregnant rats were purchased from Charles River (Calco, Italy). Rats used for *in vivo* studies were bred at a low luminance of 5–10 lux (12 h light and 12 h dark; lights on at 7 a.m.) at constant temperature (22 ± 1 °C) and relative humidity (60 ± 10%), provided drinking water and a complete pellet diet (Mucedola, Settimo Milanese, Italy) *ad libitum*, and housed under conditions of environmental enrichment in the IRCCS Ospedale Policlinico San Martino Animal Facility. All animal manipulations and procedures were performed in accordance with the guidelines established by the European Community Council (Directive 2014/26/EU of March 4, 2014) and were approved by the Italian Ministry of Health (Authorization 484/2021-PR and 357/2019-PR for the *in vitro* and *in vivo* experiments, respectively). All efforts were made to minimize suffering and reduce the number of animals by complying to the 3Rs principle.

### Synthesis, Characterization, and Stabilization of Nanoparticles

#### Synthesis and Stabilization of 4 nm PtNPs

PtNPs were prepared following a previously reported protocol.^49,51^ All reagents were prepared in ultrapure water for the reaction. Briefly, 160 μL of 0.5 M hexachloroplatinic acid (H_2_PtCl_6_, P7082, BioXtra grade, Merck) and 192 μL of 0.5 M trisodium citrate (Na_3_C_6_H_5_O_7_, 71402, BioUltra grade, Merck) were consecutively added to 80 mL of ultrapure water at room temperature (RT) with constant stirring. Then, 5.4 mL of 0.06 M sodium borohydride (NaBH_4_, 213462, Merck) was added drop by drop with stirring, and the temperature was raised to 75 °C and kept there for 30 min. The formed solution of brown-black colloidal suspension was left to cool down at RT and then washed multiple times using 2 mM sodium citrate to remove possible traces of unreacted platinum precursor using 10 kDa Amicon centrifugal filters. The final PtNP concentrated solution was adjusted to neutral pH using NaOH and diluted in a 10 mg/mL solution of albumin from rat serum (RSA, Sigma-Aldrich). Additional washes were performed to eliminate citrate excess and to concentrate back the NPs until reaching a concentration of about 1 mg/mL (and 10 mg/mL RSA). RSA-stabilized PtNP concentration was quantified by inductively coupled plasma mass spectroscopy (ICP-MS). The working concentration for intravitreal injection was 0.1 mg/mL of PtNPs with 1 mg/mL RSA (close to physiological vitreous composition).

#### Preparation of Citrate-Stabilized 5 nm CeO_2_NPs

1 mg/mL TMAOH-stabilized CeO_2_NPs (1 mg/mL) were purchased from Applied Nanoparticle (Nanotech Engineering Company).

TMAOH-stabilized CeO_2_NPs underwent ligand exchange to obtain citrate-stabilized CeO_2_NPs. The ligand exchange was performed by washing the colloidal suspension multiple times by centrifugation using 2 mM trisodium citrate and sonicating the suspension for 20 min before every centrifugal step. The NPs were resuspended to obtain a 4 mg/mL solution within a few days to avoid possible aggregation. The citrate-stabilized CeO_2_NP suspension was sonicated for 20 min prior to any measurements. The citrate-stabilized CeO_2_NP suspension was analyzed by TEM and DLS, showing improved colloidal stability and a smaller hydrodynamic radius at physiological pH values compared to TMAOH-stabilized CeO_2_NPs.

#### Dynamic Light Scattering

Nano ZS (Malvern Instruments, UK) was used to determine the hydrodynamic diameter of PtNPs by dynamic light scattering. Three independent measurements were performed with 11 runs of accumulation.

#### Agarose-Gel Assay

PtNP electrophoretic runs in the presence and absence of RSA corona were evaluated by 2.5% agarose gel, 90 V, 25 min.

#### Oxygen Sensor Measurements

The experiments were performed in 8 mL glass vials closed with a septum cap. The pressure inside the system was kept in equilibrium with the atmospheric pressure by inserting a thin needle into the septum. The variation in O_2_ % of the gas phase inside the vial was recorded with a fiber-optic needle sensor (FireSting-O_2_ sensor from Pyroscience).

The vials were filled with 300 μL of H_2_O_2_ (2.5 M), 800 μL of acetate buffer (pH = 5), and 400 μL of NP suspension to obtain final concentrations of 0.2 ppm for PtNPs and 1000 ppm for CeO_2_NP.

The reaction mixture was kept at room temperature (ca. 25 °C) for the whole experiment, and the air control (baseline) presented ca. 20% of O_2_.

### Assays of Oxidative Stress in Primary Rat Cortical Neurons

Primary cortical cultures were prepared from wild-type Sprague–Dawley rats (Charles River, Calco, Italy), and all efforts were made to minimize suffering and reduce the number of animals used. Briefly, mice were sacrificed by CO_2_ inhalation, and 18-day embryos (E18) were removed by Cesarean section. Enzymatically dissociated cortical neurons were plated on poly-d-lysine-coated (0.1 mg/mL, Sigma) glass coverslips at a total density of 80000 cells/well. Cultures were incubated at 37 °C, 5% CO_2_, and 90% humidity in medium consisting of Neurobasal (Gibco/Thermo-Fischer Scientific) supplemented to reach final concentration of 1% glutamine, 1% penicillin/streptomycin, and 5% B27 supplement (Gibco/Thermo-Fischer Scientific).

#### 2′,7′-Dichlorofluorescein (DCFDA) Assay

Primary rat cortical neurons (4 × 10^4^ cells per well) were seeded in 12-well plates and grown under standard cell culture conditions. Cells were treated with RSA-stabilized PtNPs at a final concentration of 50 μg/mL. After 48 h of incubation, neurons were washed to remove noninternalized PtNPs, and the quantification of intracellular ROS (H_2_O_2_) level was performed by a 2′,7′-dichlorofluorescein (DCFDA) assay. Neurons were incubated with 1 mM H_2_O_2_ for 15 min at 37 °C in the presence of the DCFDA probe in FluoroBrite medium (Gibco). Then neurons were washed with fresh FluoroBrite, and the DCF fluorescence intensity was measured by an Infinite 200 Pro Tecan microplate reader. The excitation filter was set at 485 nm and the emission filter at 535 nm. Results were normalized with respect to the untreated cells (negative controls). H_2_O_2_ treatment in the absence of PtNPs was used as positive control.

#### Dihydroethidium (DHE) Assay

Primary rat cortical neurons were seeded at a density of 1 × 10^4^ in a 96-well plate (Falcon) in a final volume of 100 μL and grown under standard cell culture conditions. Cells were treated with RSA-stabilized PtNPs at a concentration of 50 μg/mL for 48 h. Then neurons were washed to remove noninternalized PtNPs and incubated with 5 μM of antimycin A (ThermoFisher) for 24 h. The quantification of intracellular ROS (superoxide anions) level was performed with a dihydroethidium assay (DHE) kit (Abcam). DHE was used at a concentration of 5 μM and incubated for 1 h 30 min with cells. Fresh FluoroBrite was added before measuring the DHE intensity via an Infinite 200 Pro Tecan microplate reader. The excitation filter was set at 485 nm and the emission filter at 590 nm. Results were normalized with respect to the untreated cells (negative controls). Antimycin A treatment in the absence of PtNPs was used as positive control.

#### Apoptosis/Caspase 3/7 Assay

Primary rat cortical neurons were seeded at a density of 1 × 10^4^ in a 96-well plate in a final volume of 100 μL and grown under standard cell culture conditions. Neurons were treated with RSA-stabilized PtNPs at a concentration of 50 μg/mL. After 48 h of incubation, cells were incubated with 1 mM H_2_O_2_ for 15 min or with 5 μM antimycin A for 24 h, followed by 30 min incubation with CellEvent caspase 3/7 detection reagent (ThermoFisher). Fresh FluoroBrite was added before measuring the caspase fluorescence intensity by an Infinite 200 Pro Tecan microplate reader. The excitation filter was set at 502 nm and the emission filter at 530 nm. Results were normalized with respect to the untreated cells (negative controls). H_2_O_2_ and antimycin A treatments in the absence of PtNPs were used as positive controls.

### Transmission Electron Microscopy (TEM)

#### Imaging of PtNPs

3 μL of the colloidal sample (at an appropriate dilution) was deposited by drop-casting on a grid (CF150-Cu-50 - carbon film 150 mesh) and then dried under vacuum. A statistical NP size distribution was built by measuring the diameter of at least 200 NPs using ImageJ software.

#### Imaging of Primary Neurons

Primary rat cortex neurons were incubated with RSA-stabilized PtNPs at a concentration of 50 μg/mL for 48 h and then detached by trypsin-EDTA, centrifuged, and resuspended in a fixing solution of 2% glutaraldehyde in cell culture media under slow stirring conditions, for 45 min at RT. Neurons were then centrifuged and incubated with 2% glutaraldehyde in Na-cacodylate buffer 0.1 M with under gentle stirring for 1 h at RT and washed 3 times for 10 min with 0.1 M Na-cacodylate buffer. Neurons were then postfixed in 1% osmium tetroxide in 0.1 M Na-cacodylate buffer for 90 min. Cells were then stained overnight at 4 °C in an aqueous 1% uranyl acetate solution. After several washes in Milli-Q water, samples were dehydrated in a graded ethanol series (70%, 90%, 96%, 100% v/v) and embedded in EPON resin. Untreated cells were used as controls. Sections of 70 nm thickness were cut using a diamond knife on a Leica EM UC6 ultramicrotome.

#### Imaging of Explanted Retinas

Retinas were dissected 24 h after intravitreal injection of PtNPs. Pieces of the retina were placed in a Teflon multiwell support and quickly washed in 0.1 M Na-cacodylate buffer and then fixed with 2% glutaraldehyde in Na-cacodylate buffer 0.1 M for 2 h at room temperature (RT) and washed three times for 10 min with 0.1 M Na-cacodylate buffer. Retinal pieces were then postfixed in 1.5% osmium tetroxide and 1.5% potassium ferrocyanide in 0.1 M Na-cacodylate buffer for 90 min and washed 3 times with PBS and 2 times with water. Retinal pieces were then stained for 45 min with uranyl free acetate replacement 1% in H_2_O at room temperature in the dark. After several washes in Milli-Q water, samples were dehydrated in a graded ethanol series (70%, 90%, 96%, 100% v/v). For the embedding in EPON resin, samples were incubated first in propylene oxide for 30 min, then in EPON:propylene oxide (1:1) overnight, EPON:propylene oxide (2:1) for 3 h, and finally EPON for 48 h. Ultramicron slicing was operated using a Leica EM UC6 ultramicrotome. 1 μm slices were cut with a glass knife and stained with toluidine blue to visualize retinal layers. Once the area was selected, 70 nm sections were cut using a diamond knife. Imaging was performed with a JEOL JEM 1011 (Jeol, Japan) microscope operating at 100 kV accelerating voltage.

### Photo-oxidative Damage Procedure

To induce photo-oxidative damage, SD rats were individually placed into transparent plexiglass cages without litter or bottles of water to avoid any shadow area. Cages were placed into a cabinet (cod. 3-00001125-0, Tecniplast) from which bidirectional light of 1000 lux was provided continuously for 24 h. At the end of the photo-oxidative procedure, the low-luminance condition (5–10 lux) was restored and maintained until the next experimental procedure.

### Intravitreal Injection Procedure

1 week before (predamage treatment) or 2 days after (postdamage treatment) the photo-oxidative damage, animals were anesthetized via isoflurane inhalation (3% induction; 2% maintenance). Pupils were dilated with eyedrops of tropicamide (10 mg/mL, Visumidriatic, VisuFarma) and locally anesthetized with benoxinate hydrochloride (4 mg/mL, Novesina, Alfa Intes) eyedrops. Maintaining a 45° angle, two sequential punctures were made with a 30-gauge needle for optimal penetration of the conjunctiva. This allowed a more accessible path to be opened for a second blunt 34-gauge Hamilton needle used for injection. A microinjector (UMP3T-1, WPI) connected to the Hamilton needle via a Teflon tube was used to control the flow of the injected solution. The flow rate was 200 nL/s for a total volume of 2 μL. Surgical procedures were carried out using a manual surgical microscope (Leica M651), and the corneas were kept wet throughout the operation with a sterile saline solution. In postoperative prophylaxis, corneas were treated with tobramycin and dexamethasone (0.3%+0.1%, Tobradex, Alcon).

### Flash-Electroretinogram (fERG) Recordings

Animals were dark-adapted for 1 h, and the recordings were performed in a dark room. Under dim red light, animals were anesthetized via isoflurane inhalation (3% induction; 2% maintenance) and placed on a stereotaxic apparatus located inside a Ganzfeld dome (Retimax, CSO Florence, Italy). Pupils were dilated with eyedrops of tropicamide (10 mg/mL, Visumidriatic, VisuFarma) and locally anesthetized with benoxinate hydrochloride (4 mg/mL, Novesina, Alfa Intes) eyedrops. Corneas were kept wet throughout the recordings with a sterile saline solution. Active circular-shaped platinum electrodes were placed on the corneas, and reference and ground platinum electrodes were placed subcutaneously on the cheeks and the scalp, respectively. The body temperature was monitored with a rectal probe and kept at around 37 °C with a heating pad. Retinal responses were obtained by recording one eye at a time and closing the contralateral one. The fERG protocol involves the use of flashes with light intensities increasing on a logarithmic scale (from 0.001 to 10 cd s m^–2^). Three responses were recorded with an interstimulus interval of 5 s and then averaged. During the recording session, high-pass (0.1 Hz) and low-pass (3 kHz) filters were used. The parameters extracted from the recordings were the amplitudes of the A-wave, B-wave, and oscillatory potentials (OPs). The A-wave amplitude was measured from the baseline to the first negative peak, while the B-wave amplitude was measured from the A-wave negative peak to the highest positive peak. The OPs were measured by filtering the traces with a high-pass filter at 60 Hz, and the amplitudes and the latencies of peaks 1–4 were summed.

### High-Density Multielectrode Array (HD-MEA) Recordings

*Ex vivo* retinal recordings were performed using the a high-density multi-electrode array (HD-MEA) (BioCam X, 3Brain). Light stimuli were provided by a projector (E4500MKII, EKB Technologies Ltd.) coupled with a Z16 APO microscope (Leica, Wetzlar, Germany), to focus and center the stimulus on the retina. Animals were euthanized by CO_2_ inhalation and cervical dislocation. The eyes were enucleated and marked for a dorsal–ventral orientation. The retinas were quickly dissected in oxygenated (95% O_2_, 5% CO_2_) Ames’ medium (Sigma-Aldrich, St. Louis, MO) and divided into dorsal and ventral hemiretinas. Samples were then placed on a CMOS-based BioCam X high-density multielectrode array (Arena chip; 3Brain, Pfäffikon, Switzerland). The 4096 electrodes were directly in contact with the RGCs of the recorded hemiretinas. Peak sorting and peak detection were performed with a routine provided by 3Brain based on a Henning sorting algorithm. The sorting results were manually supervised and adjusted to eliminate any noise that was incorrectly sampled as a signal. For successive analyses, only channels with a firing rate greater than 0.5 spike/s during the entire recording were considered. The polarity of the RGCs was evaluated by the number of peaks evoked during the full-field black-and-white flashes. Forty sweeps with durations of 10, 50, and 250 ms interspersed with 4 s of gray were presented. We also analyzed the spatial resolution of the hemiretinas by using a reverting grating stimulation with spatial frequencies ranging from 0.3 to 0.8 cpd at 2 Hz and recording the spike responses to the bars. The cdp was calculated using the linear measurement 1 cpd = 64 μm as a reference.^78^

### Retina Immunostaining Procedures

Animals were euthanized by CO_2_ inhalation and cervical dislocation. Eyes were enucleated, marked for dorsoventral orientation, fixed in paraformaldehyde 4% (Sigma-Aldrich, St. Louis, MO) in 0.1 M phosphate buffered saline (PBS, Sigma-Aldrich, CO. St. Louis, MO) overnight, and rinsed three times for 10 min in 0.1 M PBS. Eyes were dissected to remove the cornea, iris, and lens. The obtained eyecups were cryoprotected by equilibration in 15% and 30% sucrose solutions, embedded in an OCT freezing medium (Tissue-Tek; Qiagen), and frozen in dry ice. Retinal slices of 25 μm were cut with a MC5050 cryostat (Histo-Line Laboratories), collected on gelatin- and polylysine-coated glass slides, and stored at −20 °C before immunostaining. For all immunohistochemical analyses, slices were rinsed three times in 0.1 M PBS to remove excess OCT and then incubated with 10% normal goat serum (NGS, Sigma-Aldrich, St. Louis, MO) for 1 h at RT to avoid nonspecific antibody binding. According to Table 1, primary antibodies against the Müller cell/astrocyte marker GFAP and the microglial marker IBA1 were diluted in 0.05% Triton X-100 in 0.1 M PBS and incubated overnight at 4 °C. Slices were rinsed three times in 0.1 M PBS and incubated with bisbenzimide nuclear labeling (1:300 Hoechst, Sigma-Aldrich, St. Louis, MO) and secondary antibodies diluted 1:100 for 1 h at room temperature. Slices were rinsed three times in 0.1 M PBS to eliminate excess antibodies and mounted with 60 × 40 mm coverslips in Mowiol mounting medium (Sigma-Aldrich, St. Louis, MO). Retinal images were acquired with a laser-scanning confocal microscope (Leica SP8; Wetzlar, Germany) with a 40× oil immersion objective. Hemiretinas were incubated with bisbenzimide nuclear labeling (1:300) for 30 min, washed three times in 0.1 M PBS, and mounted between two 60 × 40 mm coverslips.

**Table 1:** 

Primary antibody	Localization	Supplier	Cat. No.	Host	Type	Dilution	Secondary antibody	Dilution	Host
Anti-GFAP	Müller cell	Sigma	G3893	Mouse	Monoclonal	1:250	AlexaFluor 488	1:100	Goat
Anti-IBA1	Microglia	Wako	019–19741	Rabbit	Polyclonal	1:500	AlexaFluor 568	1:100	Goat

### Retina Morphometric Analyses

All morphometric analyses of the retina were performed by imaging 290.91 × 290.91 × 20 μm central and peripheral *z*-stacks with *XY* resolution of 1024 × 1024 pixels, with *Z* steps of 330 nm, of dorsoventral slices passing through the optic disk. For the hemiretinas, the acquisition parameters were 129.21 × 129.21 × 70 μm *XY* resolution of 3224 × 3224 pixels. Acquisition parameters were kept constant throughout the imaging sessions for comparison purposes. All the images were processed on ImageJ (NIH, Bethesda, MD).

#### Bisbenzimide Nuclear Staining

To evaluate the ONL thickness and the number of PRs, retinal sections were divided into 20 equidistant areas (10 dorsal and 10 ventral), taking the optic nerve as a reference. The ONL thickness was measured as the ratio between ONL and the total retinal thickness, and the number of PR rows was simultaneously calculated.

#### GFAP and IBA1 Analysis

To evaluate the GFAP integrated density, the averages of three regions of interest (ROIs; 100 × 100 pixel) for each layer (GCL, IPL, ONL) were summed.

Single IBA1-positive cells were isolated from the ONL and binarized. The circularity index was calculated using the *Circularity* plugin in the ImageJ Fiji distribution (circularity = 4π(area/perimeter^2^). A circularity value of 1.0 indicates a perfect circle. As the value approaches zero, it indicates an increasingly elongated polygon. Sholl analysis and the sum of the intersections were performed by excluding the cell body and evaluating both the extension and the number of branches intersecting concentric circles centered in the cell body with a step increase in diameter of 1.4 μm. IBA1-positive cells were manually counted in the ONL.

### Statistical Analysis

The sample size needed for the planned experiments (*n*) was predetermined using the G*Power software for the ANOVA test with four experimental groups, considering an effect size of 0.25–0.40 with α (type-I error) = 0.05 and 1-β (type-II error) = 0.9, based on similar experiments and preliminary data. Experimental data are expressed as means ± SEM throughout for *n* = sample size. The normal distribution of experimental data was assessed using the D’Agostino–Pearson normality test. To compare two sample groups, either the Student *t*-test (normal distribution) or the Mann–Whitney *U*-test (non-normal distribution) was used. To compare more than two normally distributed experimental groups, one- or two-way mixed ANOVA followed by Holm–Šídák, Tukey, or Fisher LSD multiple comparison tests was used. Cumulative distributions between experimental groups were analyzed by the Kolmogorov–Smirnov test. Contingency analysis was performed by using the Fisher exact test. Correlation tests between variables were performed based on the Pearson correlation coefficient. A *p* value <0.05 was considered significant. Statistical analysis was conducted using MATLAB R2022b and GraphPad Prism 9.5.1.

## Supporting Information Available

Additional results on the characterization of PtNPs, their presence in the outer retina, the correlation between B/A ERG waves in healthy and light-lesioned rats, the effects of the preventive treatment with PtNPs on microglia activation in light-damaged retinas, and the effects of the postlesional treatment with PtNPs on retinal ganglion cell firing and microglia activation in the ventral light-damaged retina (PDF)
