# Supplementary material for: Platinum Nanozymes Counteract Photoreceptor Degeneration and Retina Inflammation in a Light-Damage Model of Age-Related Macular Degeneration
Source: ACS Nano. 2023 Nov 7;17(22):22800–20. doi: 10.1021/acsnano.3c07517 (PMC10690844; doi:10.1021/acsnano.3c07517)
Supplement: Supplementary file 1 — nn3c07517_si_001.pdf [file nn3c07517_si_001.pdf]

Platinum nanozymes counteract photoreceptor degeneration and retina inflammation in a light-damage model of age-related macular degeneration

Sara Cupini[1,2*], Stefano Di Marco[1,3*], Luca Boselli[4], Alessio Cavalli[1,2], Giulia Tarricone[4], Valentina Mastronardi[4], Valentina Castagnola[1,3], Elisabetta Colombo[1,3], Pier Paolo Pompa[4*§], Fabio Benfenati[1,3*§]

[1] Center for Synaptic Neuroscience and Technology, Istituto Italiano di Tecnologia, Largo Rosanna Benzi 10, 16132 Genova, Italy

[2] Department of Experimental Medicine, University of Genova, Viale Benedetto XV 3, 16132 Genova, Italy

[3] Nanobiointeractions & Nanodiagnostics, Istituto Italiano di Tecnologia, Via Morego 30, 16163 Genova, Italy

[4] IRCCS Ospedale Policlinico San Martino, Largo Rossana Benzi 10, 16132 Genova, Italy

* Equal contribution

§ Corresponding authors

SUPPORTING INFORMATION

SUPPLEMENTARY FIGURES

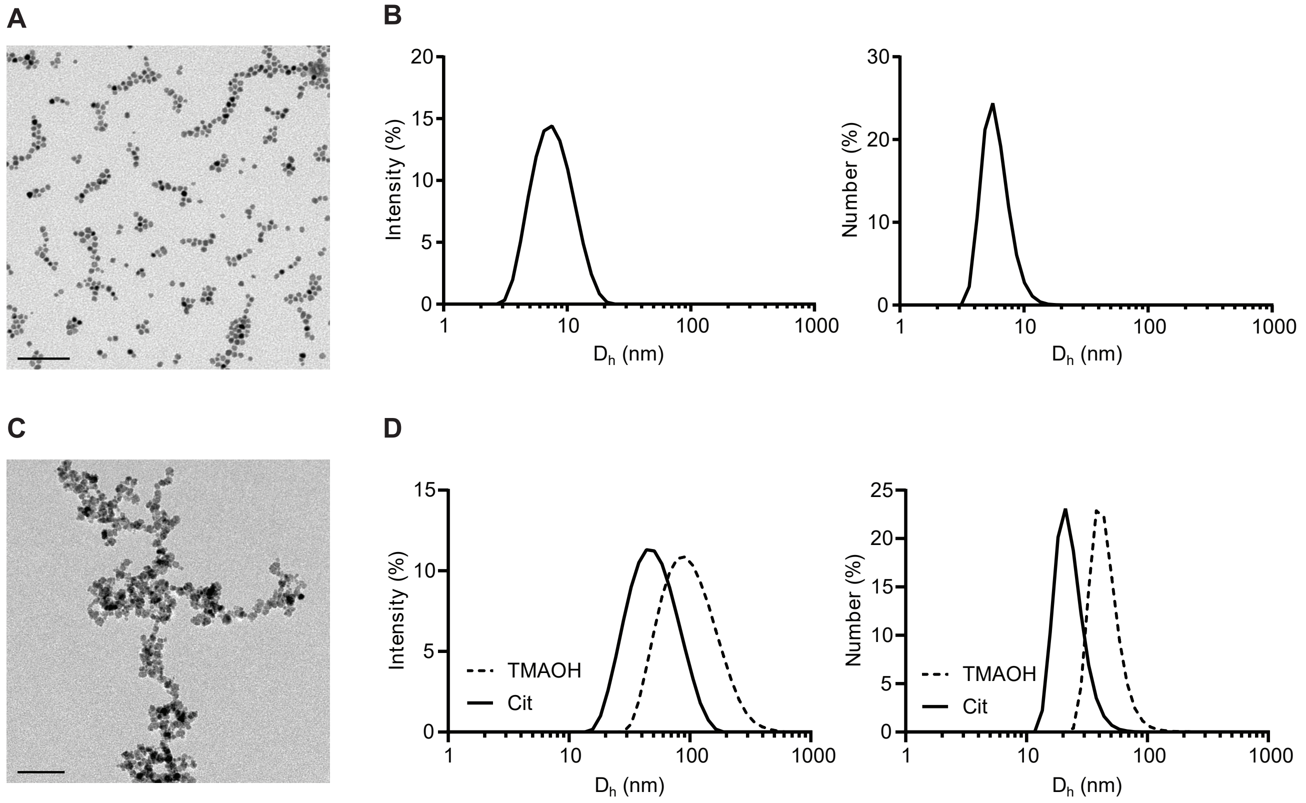

Figure S1. Characterization of **PtNPs and CeO_2NPs.**

A,B. Representative TEM micrograph (**A**; scale bar, 50 nm) of citrate-stabilized PtNPs and DLS measurement (**B**) of the hydrodynamic diameter distribution expressed as intensity (peak = 7.53 nm) and number (peak = 5.61 nm) of citrate-stabilized PtNPs. **C,D.** Representative TEM micrograph (**C**; scale bar, 50 nm) of citrate-stabilized CeO_2NPs and DLS measurement (**D**) of the hydrodynamic diameter distribution expressed as intensity and number for both TMAOH-stabilized (as purchased; broken line) and citrate-stabilized (after ligand exchange; solid line) CeO_2NPs. Citrate-stabilized CeO_2NPs (intensity peak = 43.8 nm, number peak = 21 nm) resulted better dispersed than TMAOH-stabilized CeO_2NPs (intensity peak = 91.3 nm, number peak = 37.8 nm).

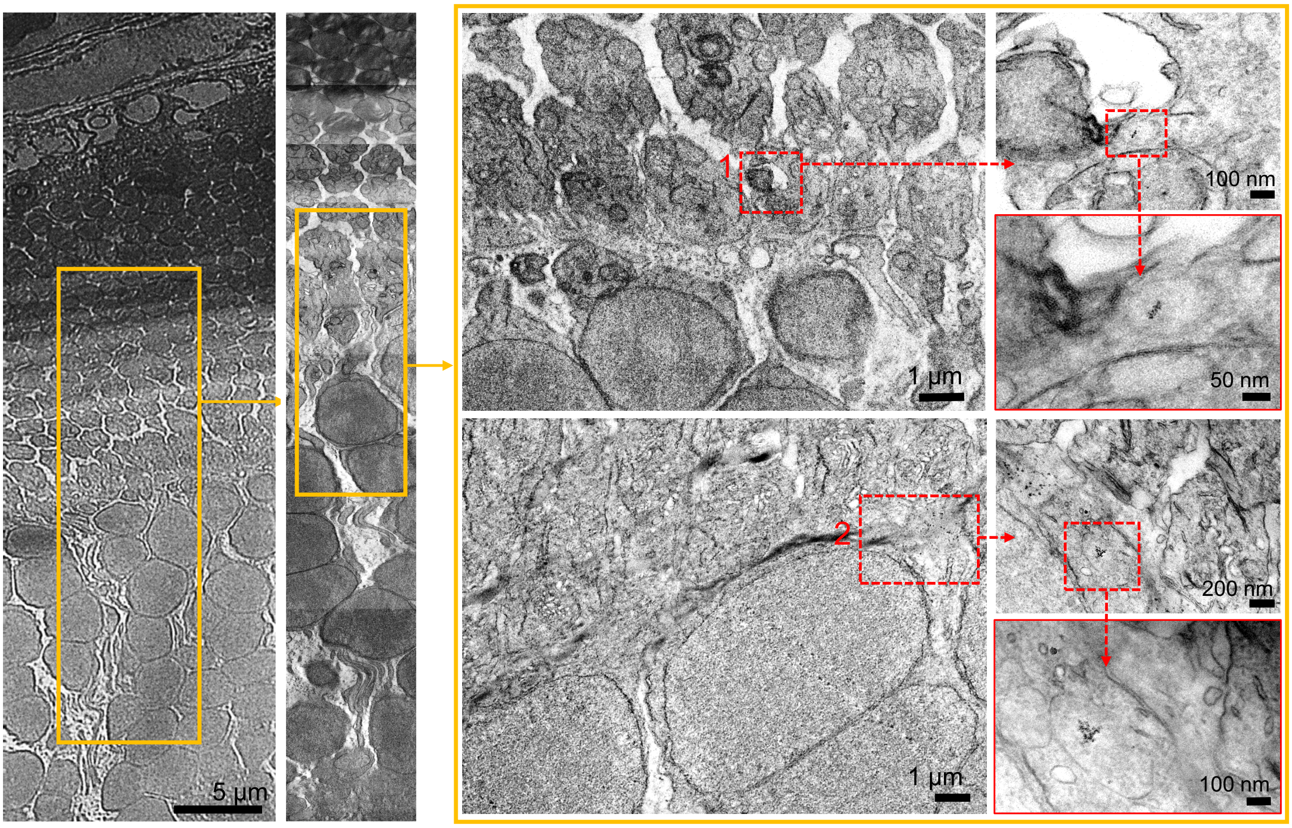

Figure S2. Transmission electron microscopy imaging of PtNP-treated retinas.
Two-month-old SD rats were subjected to the intravitreal administration of 0.1 mg/mL of RSA-coated PtNPs and the retinas explanted 24 h later. Serial magnifications of retinal ultrathin sections at the level of the ONL. From left to right, the micrographs zoom into the PR cell bodies until the appearance of high-contrast PtNPs localized in intracellular vesicles. The apparent size of the particles is compatible with the 4-nm diameter of uncoated PtNPs.

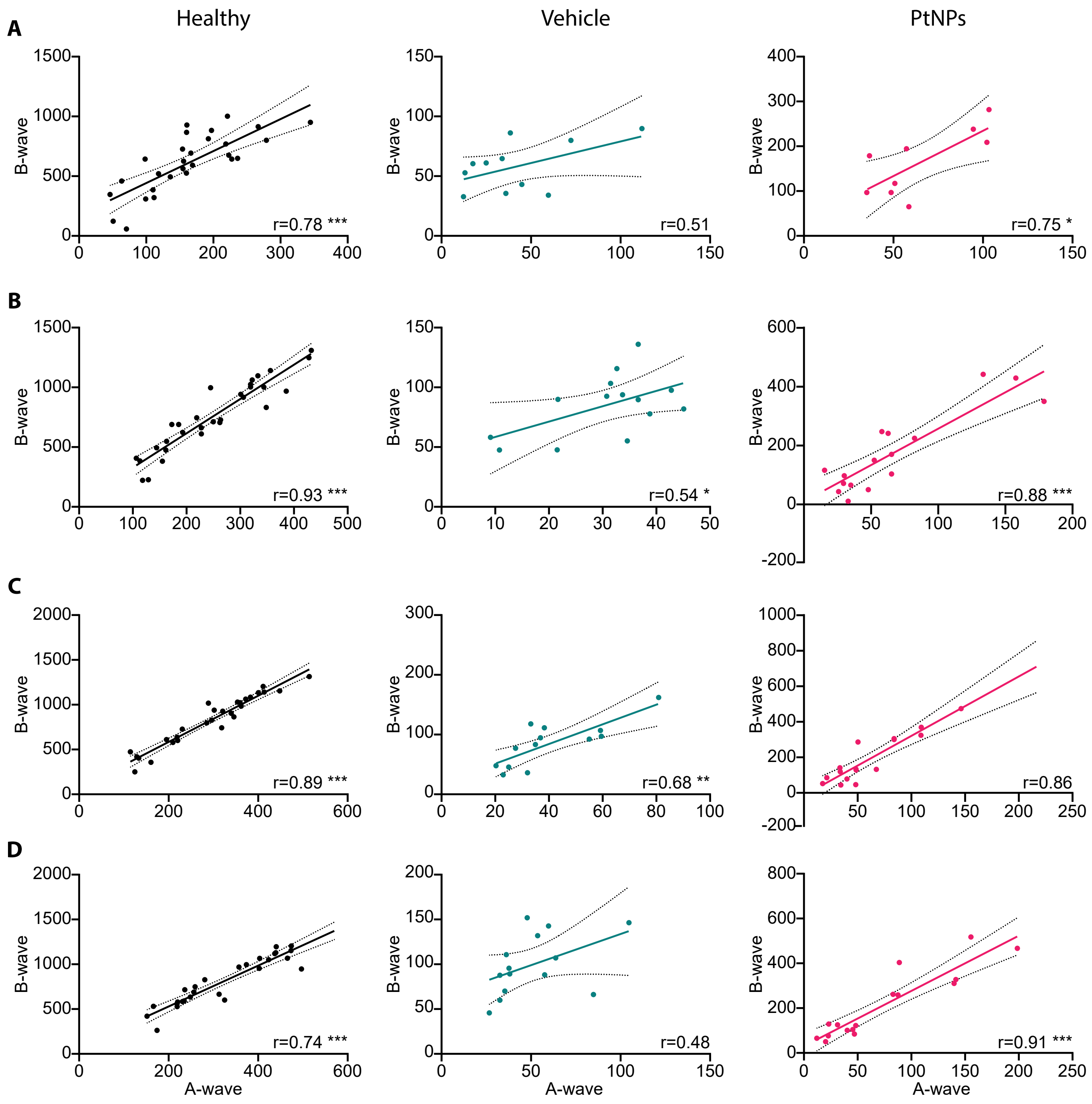

Figure S3. B/A wave amplitude correlation in ERG recordings of healthy and light-lesioned rats. A-D. The amplitude of the B-waves evoked by flash stimulation at 0.1 (**A**), 1 (**B**), 3 (**C**) and 10 (**D**) cd*m^{2}*s^{-1}. is plotted against the amplitude of the respective A-waves in healthy unlesioned rats (black; *left column*), light-lesioned rats injected with vehicle (blue; *middle column*) and light-lesioned rats injected with PtNPs (red; *right column*). The intravitreal injection was performed 7 days before the light damage and ERG recordings were performed 15 days later. For further details, see Figure 3A. The individual points were fitted using linear regression analysis. The resulting regression line, the 95% confidence intervals and the Pearson's correlation coefficient are shown in each plot.

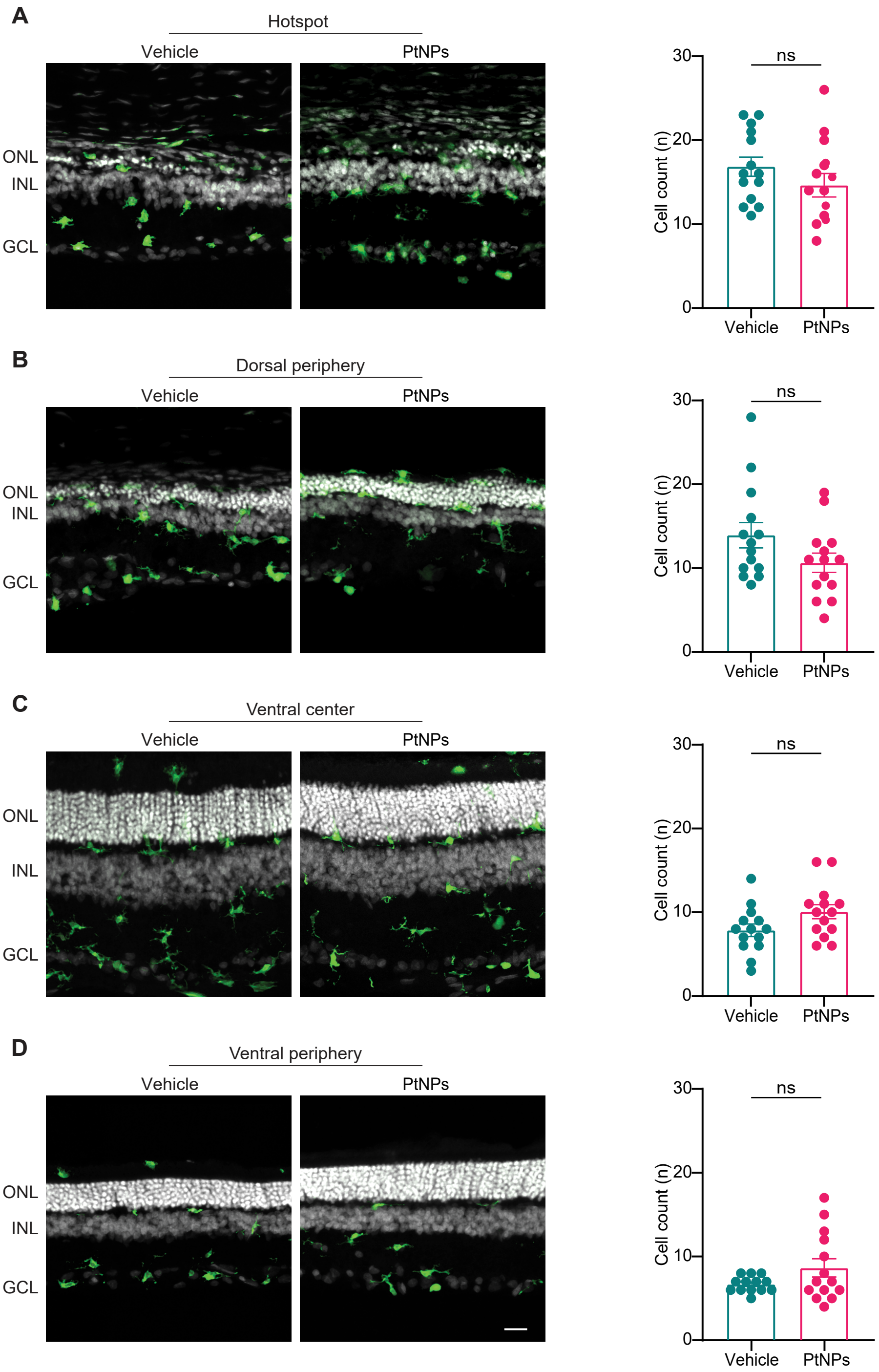

Figure S4. Effects of the preventive treatment with PtNPs on the activation of microglia in light-damaged retinas.

Retinas from animals injected with either vehicle (blue) or PtNPs (red) 7 days before the light damage were immunolabelled for the microglial marker IBA1 (green) merged with bisbenzimide nuclear labeling (white). **A-D.** *Left.* Representative IBA1-stained retinal cross-sections from dorsal hotspot (**A**), dorsal periphery (**B**), ventral center (**C**) and ventral periphery (**D**). Abbreviations: ONL, outer nuclear layer; INL, inner nuclear layer; GCL, ganglion cell layer. Scale bar, 20 µm. *Right:* Corresponding bar plots of the mean (± sem) number of IBA1-positive microglial cells counted in the ONL with superimposed individual experimental points. Sample size: n = 14 for both vehicle and PtNPs groups. ns, $p>0.05$, Mann-Whitney *U*-test/unpaired Student's *t*-test.

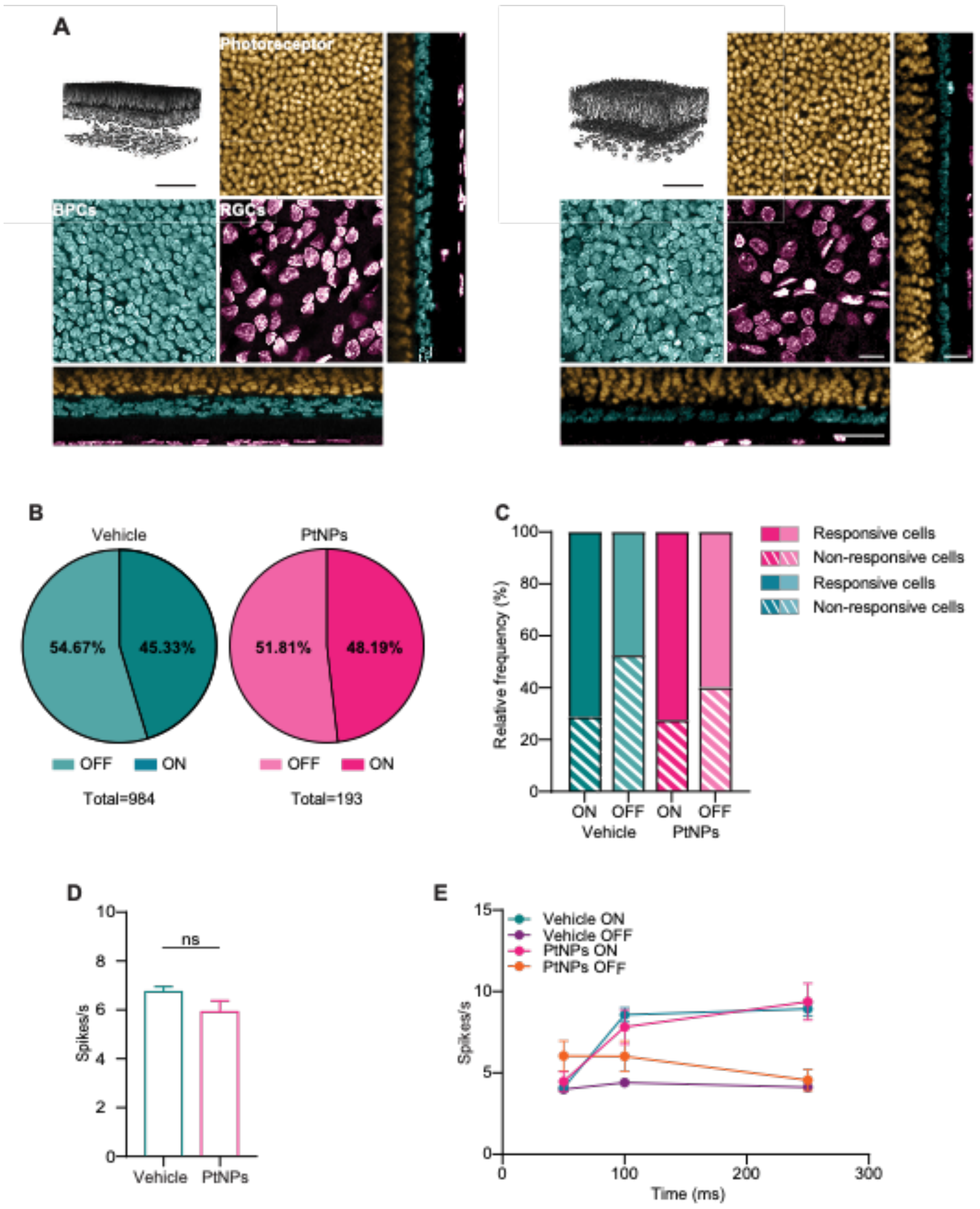

Figure S5. Effects of the postlesional treatment with PtNPs on retinal ganglion cell firing in the ventral light-damaged retina.

A. *Upper left panel:* 3D-reconstruction of representative ventral hemiretinas from vehicle (*left*) and PtNP (*right*) treated rats 24 h after the light damage (scale bar, 50 μm). *Other panels*: Z-axis magnification of each retinal layer and the orthogonal XZ, YZ projections (scale bar, 20 μm). The retinal architecture is not notably altered in both vehicle- and PtNP-treated animals. **B.** Pie charts showing the percentage of ON (dark color) and OFF (light color) RGCs on the total number of active cells recorded for each ventral hemiretina in vehicle (blue) and PtNP (red) treated rats 24 h after the light damage. In contrast to what

observed in the dorsal hemiretina, in the ventral retina the ON- and OFF-RGCs populations are comparable (p>0.05, Fisher's exact test). **C.** Percentage of responsive cells for each RGC polarity. The percentage of non-responsive cells is lower than that observed in the dorsal retina and no major treatment-dependent differences are present. **D.** Bar plots (means ± sem) of the spiking activity of total RGCs (n = 984 and 193 cells for vehicle and PtNPs, respectively) in response to a 250 msec full-field flash stimulation in ventral hemiretinas from vehicle (blue) and PtNP (red) treated rats 24 h after the light damage. No differences between the two experimental groups were observed. **E.** Temporal dynamics of RGC firing evoked by full-field flash stimulation in the ventral retina. ON-RGCs display higher dynamics of firing activity irrespective of the treatment. Sample size: 948 and 193 cells for vehicle and PtNPs, respectively. p>0.05, Mann-Whitney *U*-test (D); p>0.05, two-way mixed ANOVA.

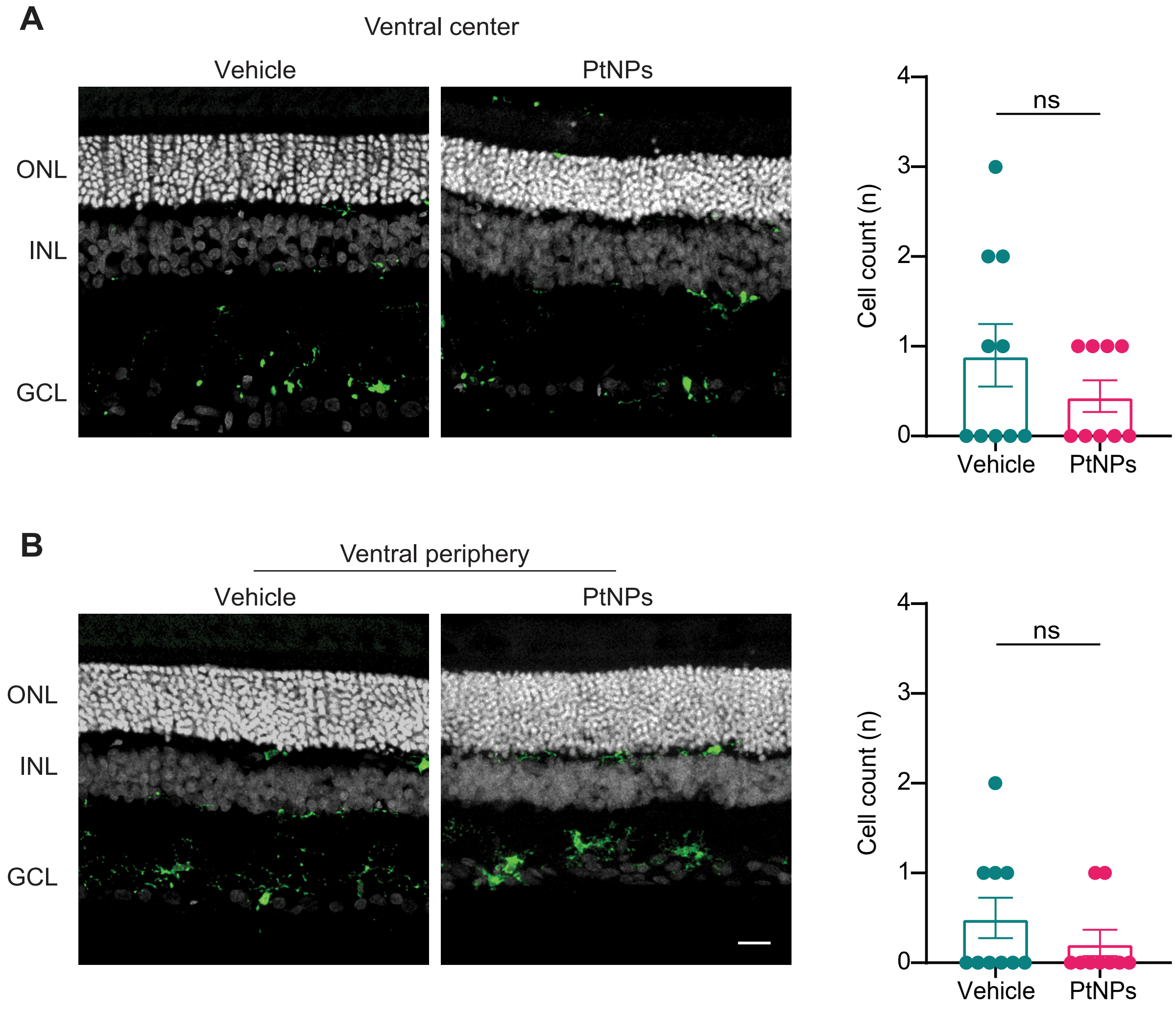

Figure S6. Effects of the postlesional treatment with PtNPs on the activation of microglia in light-damaged retinas.

Retinas from animals injected with either vehicle (blue) or PtNPs (red) 24 h after the light damage were immunolabelled for the microglial marker IBA1 (green) merged with bisbenzimide nuclear labeling (white). Abbreviations: ONL, outer nuclear layer; INL, inner nuclear layer; GCL, ganglion cell layer. Scale bar, 20 µm. **A,B.** Ventral retina center (**A**) and ventral retina periphery (**B**). Representative IBA1-stained cross-sections (*left*) and bar plots of the mean (± sem) number of IBA1-positive microglial cells counted in the ONL with superimposed individual experimental points (*right*). Microglial cells usually reside in the inner retina playing a surveillance role. Sample size: vehicle, n = 10; PtNPs, n = 9. ns, $p>0.05$, Mann-Whitney *U*-test/unpaired Student's *t*-test.

SUPPLEMENTARY TABLE

Table S1.
Linear regression analysis of the B/A wave amplitude correlation

	Healthy		Light-damaged RSA-injected		Light-damaged PtNP-injected	
Luminance	Slope	Y-intercept	Slope	Y-intercept	Slope	Y-intercept
0.1 $cd*m^2*s^{-1}$	2.65	182.49	0.36	42.96	20.2	32.49
1.0 $cd*m^2*s^{-1}$	2.87	41.17	1.28	45.65	2.47	10.52
3.0 $cd*m2*s^{-1}$	2.57	70.34	1.64	18.43	3.34	13.78
10.0 $cd*m^2*s^{-1}$	2.28	73.52	0.69	64.19	2.46	30.16

For further details, see legend to Figure S3.
